# The Influence of Hydrogen Bonding in Wood and Its Modification Methods: A Review

**DOI:** 10.3390/polym17152064

**Published:** 2025-07-29

**Authors:** Ting Zhang, Yudong Hu, Yanyan Dong, Shaohua Jiang, Xiaoshuai Han

**Affiliations:** 1 Jiangsu Co-Innovation Center of Efficient Processing and Utilization of Forest Resources, International Innovation Center for Forest Chemicals and Materials, College of Materials Science and Engineering, Nanjing Forestry University, Nanjing 210037, China; 1927957847@njfu.edu.cn (T.Z.); huyudong0123@163.com (Y.H.); 2 Institute of Environment and Sustainable Development in Agriculture, Chinese Academy of Agricultural Sciences, Beijing 100081, China; dongyanyan@caas.cn

**Keywords:** hydrogen bond, wood, organic modification, synergistic enhancement

## Abstract

Construction wood has a high economic value, and its construction waste also has multiple consumption values. Natural wood has many advantages, such as thermal, environmental, and esthetic properties; however, wood sourced from artificial fast-growing forests is found to be deficient in mechanical strength. This shortcoming makes it less competitive in certain applications, leading many markets to remain dominated by non-renewable materials. To address this issue, various modification methods have been explored, with a focus on enhancing the plasticity and strength of wood. Studies have shown that hydrogen bonds in the internal structure of wood have a significant impact on its operational performance. Whether it is organic modification, inorganic modification, or a combination thereof, these methods will lead to a change in the shape of the hydrogen bond network between the components of the wood or will affect the process of its breaking and recombination, while increasing the formation of hydrogen bonds and related molecular synergistic effects and improving the overall operational performance of the wood. These modification methods not only increase productivity and meet the needs of efficient use and sustainable environmental protection but also elevate the wood industry to a higher level of technological advancement. This paper reviews the role of hydrogen bonding in wood modification, summarizes the mechanisms by which organic, inorganic, and composite modification methods regulate hydrogen bond networks, discusses their impacts on wood mechanical properties, dimensional stability, and environmental sustainability, and provides an important resource for future research and development.

## 1. Introduction

As one of the most valuable wood products, construction wood not only creates significant economic benefits, but also its related construction waste contains secondary and even tertiary consumption value [1]. However, relying solely on increasing the amount of wood to pursue economic benefits will eventually lead to excessive destruction of forest resources. Global Forest Watch researchers using satellite data estimate that annual deforestation in the world has reached 4 million hectares, 95% of which belongs to tropical regions. Approximately 60% of deforestation is caused by livestock and agricultural development, but taking into account the third major factor, forest products (including paper and wood), this share increases to almost 75% [2]. Although wood as a renewable and recyclable material can be processed and reused at the end of its life cycle, the supply of wood resources in the wood market is far from meeting people’s rigid demand for wood, making wood resources a short-term ‘non-renewable resource’ [3]. The existence of fast-growing plantations solves the problem of a long growth cycle. However, in fast-growing wood, due to the fast growth rate, the obtained wood usually has low density, high hygroscopicity, easy deformation and cracking, and relatively poor mechanical properties [4]. In order to reduce the consumption and waste of wood and effectively improve the utilization rate of wood, researchers have processed wood to make up for or alleviate internal defects and improve its mechanical properties, such as toughness and strength [5], so as to ensure a longer service life.

The chemical composition and hierarchical structure of wood severely limit the improvement of wood mechanical properties. Over the past decade, methods to improve performance and expand applications by modifying wood have gradually matured. Common techniques include chemical modification, impregnation, and thermal treatment. After the efficient regulation of hydrogen bonds, wood is formed into a more ordered and stable system network that shows excellent results in the process of resisting external loads, and wet strength and viscosity are also significantly improved [6,7,8]; internal bonding techniques and structural elements of wood are regulated, relying on the action of hydrogen bonds to show the effect of improving the structural stability of materials [9,10,11]; this characteristic is very important for the prospect of the application of functional materials [12,13] and is also closely related to higher engineering requirements.

The concept of hydrogen bonds emerged at the beginning of the 20th century, but it has faced difficulties in theoretical and practical development. In 2011, the International Union of Pure and Applied Chemistry (IUPAC) proposed a new definition of hydrogen bonding, according to which the H atoms in X-H in a molecule or fragment (the electronegativity of X is stronger than that of H) would be attracted to atoms and groups in the same or different molecules, and in this case, this should be the basis for the formation of a hydrogen bond. This definition expands the scope of the traditional donors and acceptors of hydrogen bonds. In the past, donors mostly contained F-H, O-H, and N-H [14], while traditional acceptors were usually electronegative atoms such as F, O, and N. Later studies have shown that the donor can also be extended to some new groups, such as C-H and the H atom in the metal hydride (which forms a dihydrogenic bond), and other elements of the periodic table, which can be used as acceptors of hydrogen bonds, such as P, S, Cl, Se, Br, I, etc., and contain transition metal atoms with π-electron systems or other states that can be used as bridge elements [15,16,17,18]. Based on the IUPAC definition, we explore the radiation range that affects the expansion of hydrogen atoms and their applications in the fields of fuel and chemistry.

There is an exponential relationship between the number of hydrogen bond donors and receptors that determines the binding strength of hydrogen bonds [19,20,21]. The number and location of hydrogen bond receptors affect the potential binding pattern. When multiple bonding modes coexist, the hydrogen bond donor determines the position of the N-H group to select the best bonding mode [22]. An in-depth study of hydrogen bond suppliers/acceptors can help improve the efficiency of wood modification with hydrogen bonds. For instance, the C-H methyl hydrogen bond can stabilize the binding conformation of the double bond, and the halogen bond receptor enhanced by the C-H hydrogen bond has a higher efficiency [23]. In addition, the molecular model of its hygroscopic region is closely related to the hydrogen bond functional group, and its hygroscopic region contains at least one OH group and two hydrogen bond functional groups comprising a hydrogen donor/acceptor pair. When an isolated hydroxyl group is in close proximity to another hydrogen-bonding functional group, a new pair of adsorption sites is formed [24]. This is related to the presence of the presence of hydrogen bond donors/acceptors affects the accessibility and reactivity of cellulose fibers. Solvents with high hydrogen bond receptor strength (such as acetic acid) can form hydrogen bonds more effectively with functional groups such as hydroxyl groups in the fibers, thereby penetrating deeper into the fiber cell walls, resulting in more significant fiber expansion and increasing the grafting density of subsequent polymerization reactions. Successful grafting not only improves the thermal stability of fibers but also reduces the adsorption of water [25]. Hydrogen bond donors and hydrogen bond acceptors can also be used to prepare deep eutectic solvents (DESs), and their types and molar ratios have a great influence on solvent properties such as polarity, dipole/polarizability, hydrogen bond acidity, and hydrogen bond basicity of DESs [26]. Hydrogen bond donors (HBDs) (such as urea and carboxylic acid) and acceptors (HBAs) (such as choline chloride) form new hydrogen bond networks with wood components (such as -OH in cellulose/hemicellulose and phenolic hydroxyl in lignin). This interaction not only destroys the original hydrogen bond structure of wood (such as O6-H···O3’ between cellulose chains) but also promotes the dissociation of components by reducing the energy barrier of lignin–carbohydrate complexes (LCCs). Thus, the chemical composition and surface structure of wood are changed, which further affects the physical and chemical properties of wood [27].

**Figure 1:**
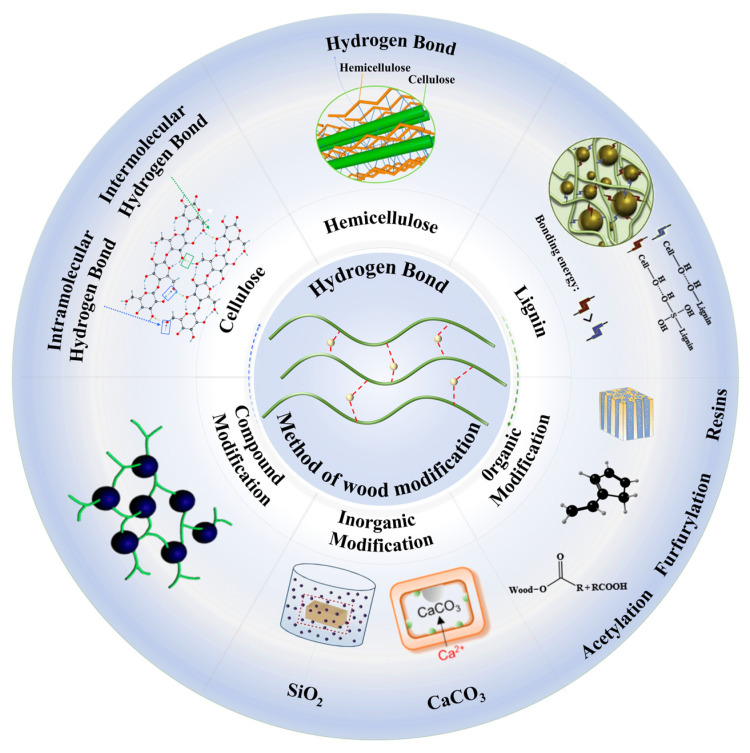
Location of hydrogen bonding in wood and modification methods [28,29,30,31,32,33,34].

This paper aims to provide thinking guidance for researchers interested in wood modification, especially the use of chemical bonds such as hydrogen bonds to improve wood properties. We discussed the formation of hydrogen bonds from the perspective of wood composition. We focused on the effects of organic and inorganic modifications on hydrogen bonds and their effects on enhancing wood strength and viscosity. Through an in-depth study of the hydrogen bond regulation mechanism, we emphasized the synergistic enhancement of hydrogen bonds and chemical bonds (Figure 1). In the conclusion, we discuss the optimization and improvement of wood-processing technology and propose corresponding solutions based on the latest research results.

## 2. The Interaction of Hydrogen Bonds in Wood Components

The cell walls of wood mainly consist of cellulose, hemicellulose, and lignin. The structural stability of wood relies on the interactions among various components, with hydrogen bonds playing an extremely important role. The dynamic properties of the hydrogen bond network are the key to regulating this function. Therefore, this section analyzes the specific role of hydrogen bonds from three perspectives: between cellulose and cellulose, between cellulose and hemicellulose, and between cellulose and lignin.

### 2.1. Hydrogen Bonds Between Cellulose Molecules

Cellulose is a polymer composed of β-(1-4)-linked glucans in a double-helix conformation, i.e., the glucose unit is flipped 180π with respect to the neighboring unit [35,36]. Hydrogen bonds are critical for the structural behavior of cellulose, particularly in maintaining the integrity of its hierarchical architecture. However, while some studies argue that there is no direct evidence for hydrogen bonds being the primary driver of cellulose fiber helical coiling at the molecular level, they consistently emphasize the necessity of an intact hydrogen bond network for this process at the supramolecular scale. For instance, the absence of intramolecular hydrogen bonds between O_2_ and O_6_ would render stepwise right-hand helical distortion unattainable [37]. Furthermore, during the preparation of regenerated cellulose fibers, the formation of hydrogen bonds is crucial for the structure and properties of the fibers [38]. Optimizing the hydrogen bond distribution in regenerated cellulose fibers by adjusting the hydrogen bond donors and hydrogen bond acceptors in the dissolution system can improve their physical strength. Meanwhile, the formation and breakage of hydrogen bonds affect the crystallinity and thermal stability of cellulose. The glass transition temperature and the initial temperature of the thermal decomposition of regenerated cellulose fibers can be increased by hydrogen bonding [39]. The crystallinity and grain size of cellulose nanofibers are also highly correlated with the evolution of the hydrogen bonding network structure [40]. By treating cellulose nanofibers with three types of DESs, three types of hydrogen bonds in cellulose nanofibers were identified: O6-H⋅⋅⋅O3’, O3-H⋅⋅⋅O5’, and O2-H⋅⋅⋅O6’. This bonds undergo a strict sequential change in the relative content and position. At the same time, the crystallinity and grain size of cellulose nanofibers increased. However, the study discussed less about other property changes in cellulose nanofibers before treatment, which somewhat limited its comprehensive evaluation of the treatment effect [41].

**Figure 2:**
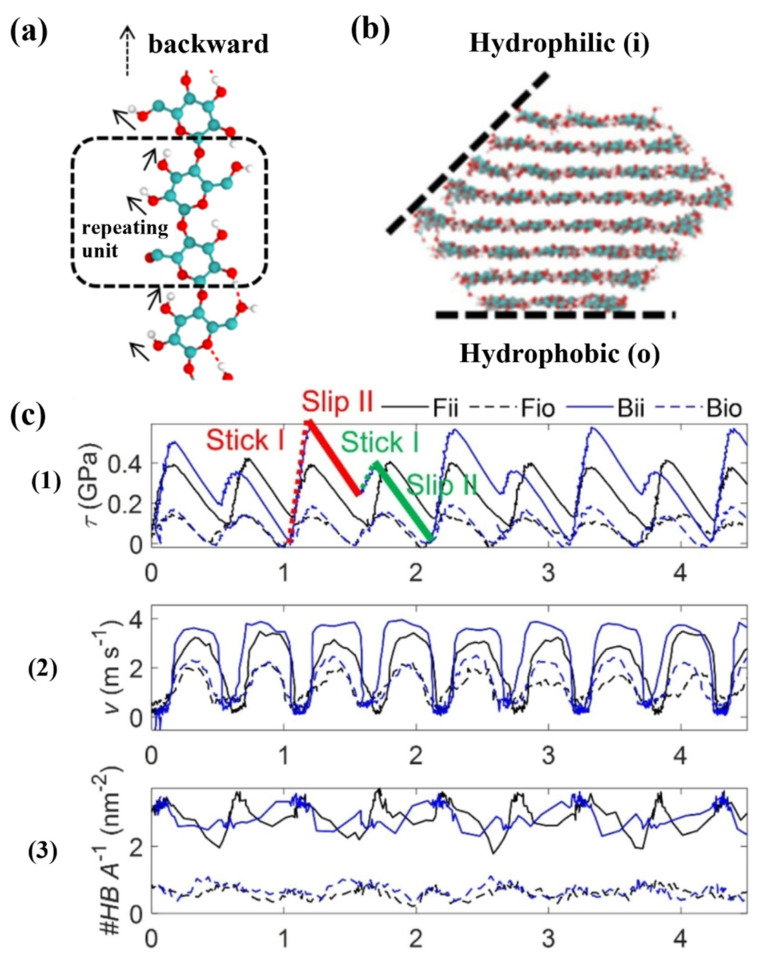
(**a**) The glucosyl unit is flipped 180 π relative to its neighboring units, indicated by the dashed square, and the black arrow indicates the preferred orientation of the hydroxyl group. (**b**) Cellulose–cellulose hydrogen bonds interacting between the hydrophilic (i) and hydrophobic (o) planes of CNCs. (**c**) Hydrogen bond densities of hydrophilic–hydrophobic configurations (Fio-Bio) and hydrophilic–hydrophilic configurations (Fii-Bii) (**c1**). Velocity of movement of crystals in stick–slip motion (**c2**). Maximum shear stress of the system (**c3**) [43]. Stick I, slip I, indicated in red, stick II and slip II, indicated in green.

In addition to discussing the significance of hydrogen bonding on the structure and properties of cellulose, some researchers have also explored the effect of hydrogen bonding on the cellulose interface [42]. The dashed box in (Figure 2a) represents this repeating glucose unit. The stable crystal structure of crystalline cellulose is an assemblage of glucose chains bound together by intermolecular interactions. That is, each monomer has one interchain hydrogen bond and two intrachain hydrogen bonds. The black arrows indicate the preferred orientation of the hydroxyl groups. Zhang et al. suggested that cellulose–cellulose hydrogen bonding plays an important role in the interactions between the hydrophilic (i) and hydrophobic (o) planes (Figure 2b) and the (110) and (200) planes of the CNC, and revealed a close connection between the hydrogen bonding areal densities and the interfacial shear stresses and mechanical stiffnesses. The hydrogen bonding densities of the hydrophilic–hydrophobic configurations (Fio-Bio) are lower than those of the hydrophilic–hydrophilic configurations (Fii-Bii) (Figure 2(c1)). As a result, the Fio and Bio systems exhibit lower maximum shear stresses (Figure 2(c3)) and the crystals move at lower velocities in stick–slip motion (Figure 2(c2)). At the same time, the hydrogen bonding surface densities of different systems vary at different stick–slip stages. When the interaction of hydrogen bonds between the cellulose nanocrystal (CNC) weakens, the shear voltage at the interface is significantly reduced. When the structure of the CNC interface does not match, the hydrogen bond density and the contact area decrease, and the shear voltage at the interface also decreases [43]. Han and other co-authors propose that hydrogen bonds have a dynamic bonding-dissociation mechanism. By adjusting the humidity and compression conditions, effective interphase coupling of cellulose nanophase fibers can be achieved, which greatly improves Young’s modulus and strength and viscosity in destroying the modified wood. The role of water molecules as bridges for enhancing hydrogen bonds has been discovered. The formation of hydrogen bond networks decisively determines the increase in the strength of the material [44].

Hydrogen bonds are also considered the main force by which cellulose chains connect to each other [45]. In the process of the compaction of cellulose nanofiber, hydrogen bonds help establish close bonds between the fibers, and various drying methods (air drying leads to a loss of moisture, resulting in the strongest hydrogen bonds, compared to drying with solvent replacement, and freeze drying reduces the form of hydrogen bonds) regulate the formation of hydrogen networks that in turn affect the mechanical properties of the material itself [28].

**Figure 3:**
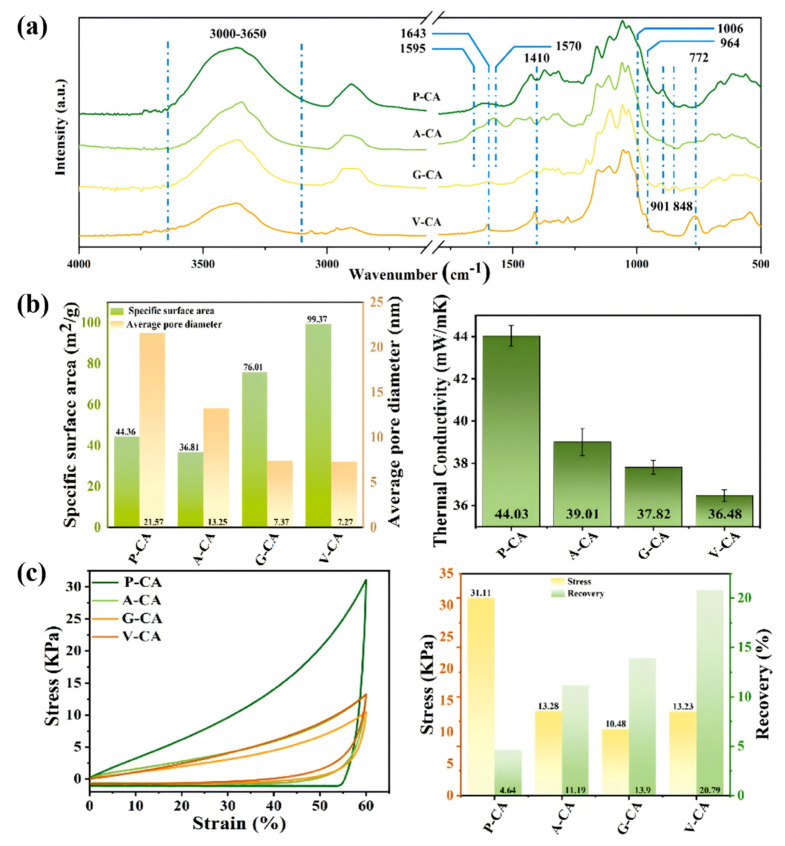
(**a**) Infrared spectra of P-CA, A-CA, G-CA, and V-CA. (**b**) Specific surface area (left) and thermal conductivity (right) of nanocellulose aerogels. (**c**) Compression Stress (left) and Compression Resilience (right) [47]. Pure nanocellulose aerogels (P-CA), 3-aminopropyltriethoxy-silane-nanocellulose aerogels (A-CA), 3-Glycidyloxypropyltrimethoxysilane-nanocellulose aerogels (G-CA), and vinyltrimethoxysilane-nanocellulose aerogels (V-CA).

Hydrogen bonds play an important role in the properties and functions of cellulose. Cellulose fibers improve the efficiency of the proton conductivity of the fiber, and hydrogen bonds play a key role. By creating a hydrogen bond network, it is possible to transfer protons, and acid–base and moisture pairs linked by the hydrogen bond can speed up the process of proton exchange, which opens up a new direction for the development of efficient proton-conducting materials [46]. By adjusting the strength of the hydrogen bond, the microstructure of the nanocellulose gel can be changed, and notice that in all samples, there is a wide range in the proton region (Figure 3a). Pure nanocellulose aerogel (P-CA) has high activity and contains the same amount of free hydrogen and oxygen bases as carbon nanosurfaces such as 3-aminopropyltriethoxy-silane-nanocellulose aerogels (A-CA). If the internal and external hydrogen bonds are removed, the strength is significantly reduced. In this case, the degree of cellulose nanofiber (CNF) aggregation is significantly reduced, and the actual specific surface area and elasticity of the nanofibric aerogel under compression are increased (Figure 3b,c), and the thermal properties are reduced, thus improving the performance of the nanofibric components in various application scenarios, such as thermal insulation and thermal processing application for the repair of load-bearing structures and environmental protection ability. Previous studies have shown that the detailed regulation of microstructure by gravity is one of the important means for the realization of potential applications in grinding and other fields [47,48].

Some scholars have pointed out that there is often an over-reliance on hydrogen bonding to explain the properties of cellulose and its materials, when in fact hydrogen bonding is only one of many molecular interactions. Wohlert analyzes the role of hydrogen bonding at several scales, including the molecular level, the intermolecular level, and the fiber level. He argues that at the molecular level, hydrogen bonds play an important role in maintaining the conformation of the cellulose molecule, but in aqueous solution, these hydrogen bonds are replaced by water molecules. At the intermolecular level, while hydrogen bonding contributes to the formation of cellulose intermolecular crystal structures, the stability of the crystals depends more on effective molecular stacking. At the fiber level, the axial modulus of elasticity is often associated with hydrogen bonds, but in reality, the role of hydrogen bonds is relatively limited. Another study discussed the controversy over the solubility of cellulose and emphasized that hydrophobic interaction dominates the properties of cellulose insoluble in water, rather than depending solely on the effect of hydrogen bonds [49].

In summary, hydrogen bonds play an indispensable role in cellulose and related fields. They not only exist between cellulose molecules but also play a key role between cellulose and other components, such as limiting the movement of molecular chains and regulating material properties. Therefore, they need to be understood in the context of other molecular forces and environmental factors.

### 2.2. Hydrogen Bonds Between Cellulose and Hemicellulose

In hemicellulose, various monosaccharide residues are linked by hydrogen bonds, forming a complex polysaccharide chain structure [50]. Xylanose is a component of hemicellulose found in abundance in the cell walls of plants. It is bound to the cellulose surface by hydrogen bonds, forming a cellulose network, and the adsorption of cellulose fibers is enhanced by the formation of hydrogen bonds [51]. This leads to hydrogen bonding, reducing the effect of removing hemicellulose, but it also has an effect on cellulose crystallinity [52]. Compared to intermolecular hemicellulose, the strength of the hydrogen bond between the cellulose surface and the hemicellulose is stronger. So, the situation of change mainly occurs in the hemicellulose matrix. When the hydrogen bond is periodically separated and renewed at this interface, stress turbulence occurs, which in turn contributes to sticky slip [53].

The hydrogen bond is an important component of the weak interaction between deep eutectic solvents (DESs) and hemicellulose, which can affect its strength and stability. DESs built on different hydrogen bond receptors also possess different methods of dissociating hemicellulose. For instance, GuHCl as the DESs for HBA synthesis exhibits superior hemicellulose solubility compared to ChCl as the DESs for HBA synthesis. This is because Cl, as a strong hydrogen bond acceptor, preferentially binds to the acetyl group in hemicellulose and weakens its hydrogen bond connection with cellulose (O5-H···O3’). Hemicellulose is an important component of the wood structure and contributes to maintaining the overall strength of the wood. The dissociation of hemicellulose may lead to a decrease in the tensile strength, compressive strength, and bending strength of the wood. Moreover, after the dissociation of hemicellulose, the toughness of the wood may be affected, manifested as being more prone to breaking or fracturing, which may limit the use of wood in certain applications that require high toughness [54]. In addition, in composite materials containing hemicellulose, hydrogen bonds also perform important regulatory functions. For example, it can affect the elasticity and plastic deformation of bacterial cellulose and hemicellulose composite films; the stronger the hydrogen bond network, the greater the elasticity of the composite film; if the network weakens, the greater the plasticity [55]. In addition, hydrogen bonds at high temperatures can also prevent the formation of molecular chain activity in high-temperature media and slow down the rate of decomposition of composite films, thereby increasing their resistance to high-temperature stabilization effects [56]; especially in the case of synergistic thermal reactions of semi-fibrous materials, hydrogen bonds can control the reaction pathway, stabilize the transition situation, reduce the barrier activation of reactions and affect the arrangement of the components that form the acid in the product and other operational characteristics [57]. However, it should be noted that under high-temperature or high-humidity conditions, the hydrogen bond-dominated stabilization effect may be replaced by hydrophobic aggregation or Van der Waals force, resulting in a decrease in hemicellulose–cellulose interface strength.

**Figure 4:**
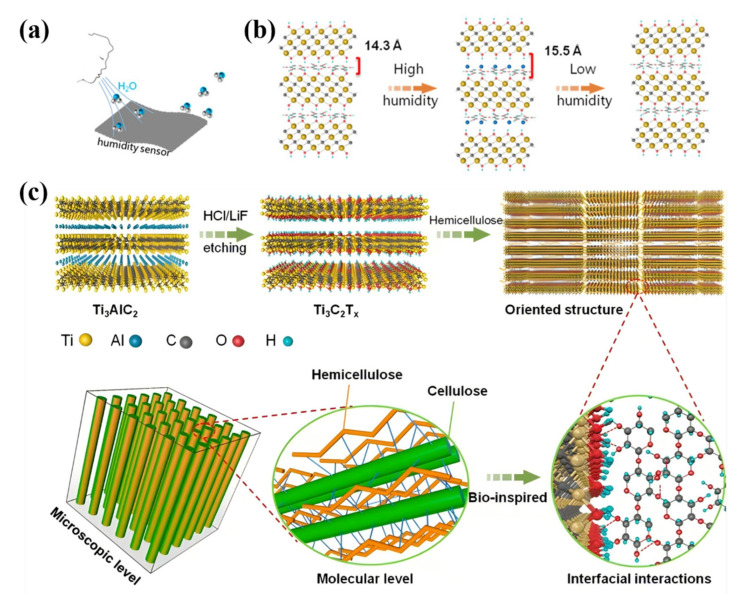
(**a**) Humidity response performance. (**b**) A large number of water molecules in the MXene–hemicellulose composite membrane is absorbed into adjacent MXene nanosheets as a function of relative humidity, and the layer spacing and resistance of the membrane increase. (**c**) Modification mechanism of MXene-half-cellulose composite membrane [29].

Compared to cellulose and lignin, hemicellulose has been relatively underexplored; however, this does not diminish its scientific significance. Inspired by the structural properties of wood, Chen et al. developed a composite material integrating wood mass and hemicellulose. Hemicellulose exhibits exceptional moisture-absorption capabilities, enabling the composite film to respond swiftly to variations in humidity. The electrical signals of the TICC Ert_x_–hemicellulose composite membrane were monitored across different relative humidity levels, highlighting the membrane’s characteristics in relation to moisture (Figure 4a). Within this composite membrane, as relative humidity fluctuates, numerous water molecules are adsorbed onto adjacent MXene nanosheets This adsorption results in increased interlayer distance and resistance within the film (Figure 4b). Consequently, this composite film can effectively function as an electrode in both supercapacitors and moisture sensors. In the MXene–hemicellulose composite film, hemicellulose serves as a molecular binder, utilizing hydrogen bonding to securely adhere adjacent MXene nanosheets together. This hydrogen bonding mechanism not only enhances the interaction between the MXene nanosheets but also promotes their organized alignment within the composite material, thus establishing a resilient layered structure. Additionally, the incorporation of hemicellulose increases the interlayer spacing between the MXene nanosheets. This hydrogen bonding plays a pivotal role in ensuring an even distribution of hemicellulose molecules between the MXene layers, thereby precisely regulating the interlayer spacing. This regulation facilitates the rapid transmission of ions, leading to a marked improvement in the electrochemical performance of the composite material (Figure 4c) [29].

### 2.3. Hydrogen Bonds Between Cellulose and Lignin

The hydrogen bond will affect the performance and use of lignin substrate. It affects the structure and final performance of lignin together with the three-dimensional structure of lignin. Lignin molecules are tightly bound to cellulose through hydrogen bonds, which further strengthens the wood cell wall structure and improves mechanical strength [58,59].

**Figure 5:**
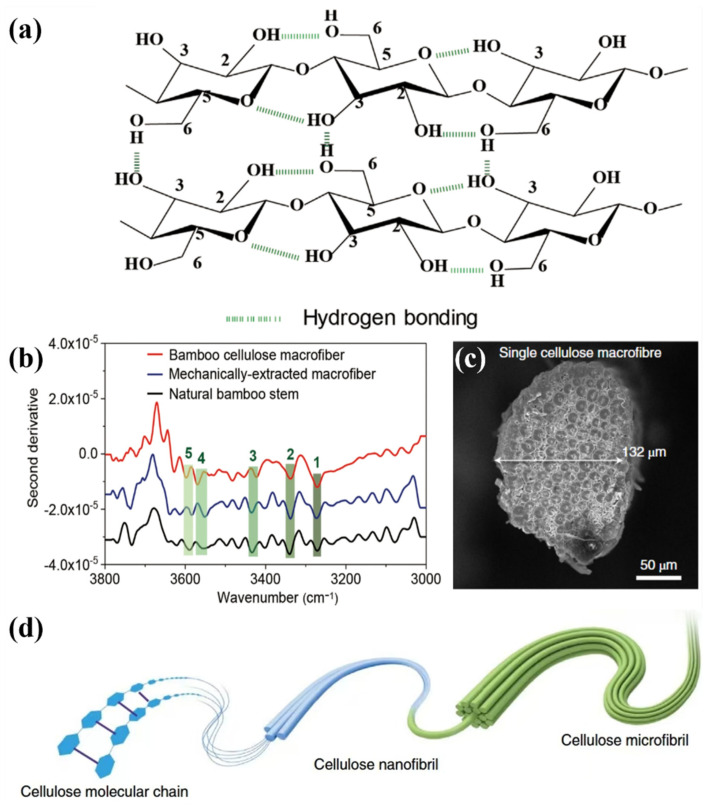
(**a**) Lignin is tightly bound to the angular region of the cell through hydrogen bonding and other covalent bonding interactions. (**b**) Second-order derivative spectra of natural bamboo poles, mechanically extracted crude fibers, and bamboo cellulose crude fibers at 3270, 3340, 3433, 3560, and 3590 cm^−1^. (**c**) Bamboo cellulose crude fiber densification. (**d**) Highly ordered structure formed by densification of the crude fibers of bamboo cellulose [60].

In wood, lignin binds to semi-crystalline cellulose through hydrogen bonding. Lignin is tightly bound to the angular regions of the cell through hydrogen bonding and other covalent bonding interactions (Figure 5a). In bamboo, the second-order conductance spectra of natural bamboo poles, mechanically extracted crude fibers, and crude fibers of bamboo cellulose at 3270, 3340, 3433, 3560, and 3590 cm^−1^ are shown in Figure 5. There are five bands at 3270, 3340, 3433, 3560, and 3590 cm belonging to the intermolecular hydrogen bonding O6-H⋯O3’ (peak 1), O3-H⋯O5’ of intramolecular hydrogen bonding (peak 2), O2-H⋯O6’ (peak 3), free O(2)H (peak 4), and free O(6)H (peak 5) (Figure 5b). The complete removal of lignin and the partial removal of hemicellulose effectively exposed more hydrophilic hydroxyl groups on the surface of cellulose nanofilaments. During the air-drying process, water gradually evaporated from the inside of the cell wall to the surface. Hydrogen bonds formed between the hydroxyl groups of bamboo fibers can promote the densification of bamboo cellulose crude fibers (Figure 5c) to form a highly ordered structure (Figure 5d) [60]. Lignin has significant UV–visible absorption characteristics, and its removal can significantly reduce the light absorption of wood. Subsequently, with the penetration of polymers such as epoxy resin or polymethyl methacrylate (PMMA), a new hydrogen bond network can be constructed between the cellulose hydroxyl group and the polymer chain segment, thereby significantly enhancing the interfacial compatibility and optical properties. The further expansion and strengthening of the hydrogen bond network can also synergistically improve the overall mechanical properties of the material [61,62].

**Figure 6:**
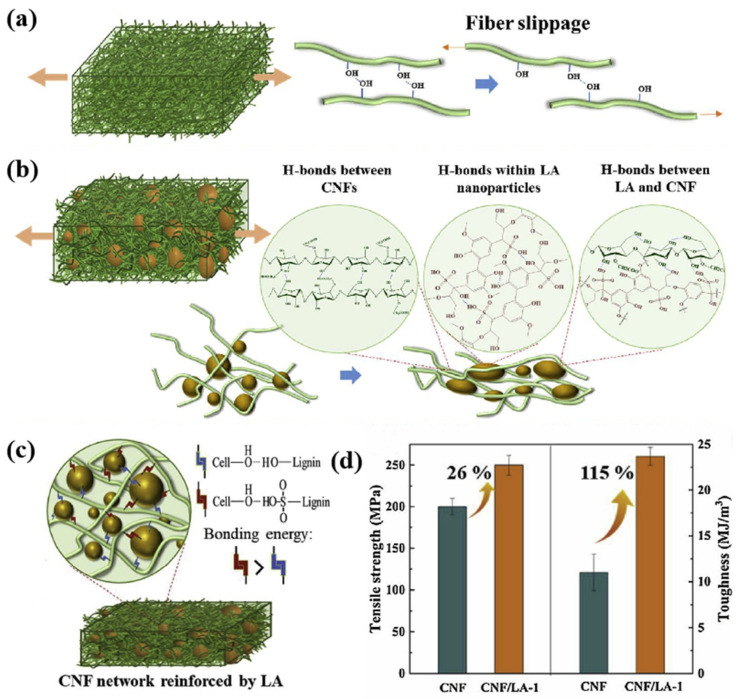
(**a**) Adjacent CNFs are prone to re-form hydrogen bonds during fiber sliding. (**b**) Cascade formation and reorganization of hydrogen bonds between adjacent CNFs, between adjacent LA molecular chains, and between LA nanoparticles and CNFs. (**c**) LA forms stronger interaction with nanocellulose. (**d**) Changes in nanocellulose toughness after the addition of 1 wt% LA [30].

The key to strengthening films made of pure cellulose nanofibers (CNFs) is that neighboring CNFs easily rearrange hydrogen bonds, and a lot of energy is consumed when the fibers slide (Figure 6a). Inspired by the structure of the lignin–cellulose compound, Zhou et al. used purified lignin sulfate (LA) as a strengthening agent to obtain a more durable compound with nanocellulose. These two elements increase hydrogen-bonding density, which contributes to the strengthening of hydrogen bonds (Figure 6c), causing physical stitching. Cascades of hydrogen bonding and recombination between neighboring CNFs, between neighboring molecular chains of LA, and between LA and CNF nanoparticles significantly contribute to the sliding deformation of neighboring CNFs and the energy loss of LA nanoparticles upon their rupture (Figure 6b). When the film is exposed to external energy, the hydrogen bond between the lignin sulfate submicrospheres and the nanocellulose breaks down as a consumable bond and dissipates some of the energy, which increases the strength of the film to some extent. The measurement results show (Figure 6d) that the addition of only 1% by weight of lignin sulfate increases the viscosity of nanocellulose from 11.0 ± 1.3 MJ/m^3^ to 23.6 ± 1.3 MJ/m^3^, and the strength increases from 198 ± 9 MPa to 249 ± 6 MPa [30]. During the decomposition of lignin, the formation of a hydrogen bond can lower the energy barrier of O-H bond destruction, facilitating the activation of the O-H bond in visible light to cause the destruction of the C-C bond and increase the efficiency of lignin decomposition [63,64].

This is not inconsistent with the mechanism by which lignin maintains its spatial conformation through hydrogen bonds and is similar to the mechanism by which lignin dissolves in ionic solutions. Both are based on the interaction of external ions with functional groups, such as hydroxyl groups in lignin molecules, to form hydrogen bonds. In lignin dissolution, the DES component competes with intramolecular hydrogen bonds (such as hydrogen bonds between lignophenol-OH and cellulose O6-H) through stronger donor–acceptor interactions. Chloride ion (Cl^−^) in choline chloride, as an effective HBA, forms hydrogen bonds with the hydroxyl groups of lignin, while urea destroys the hydrogen bonds of hemicellulose–cellulose by multi-point attachment. This will weaken the cohesion of lignocellulose and promote the separation of components [65,66,67,68]. The presence or dynamic changes in hydrogen bonds will affect the properties of some lignin materials. For example, in the production of lignin-based waterborne polyurethane, the presence of lignin helps to form a new hydrogen bond network. The structure of this network is dynamic and multi-layered. When stretched, the material consumes energy by breaking and reorganizing hydrogen bonds, which increases the strength and durability of the material [69].

The three major components of wood (cellulose, hemicellulose, and lignin) are closely cross-linked by hydrogen bonds, and their roles are not limited to the aforementioned functions, but also profoundly affect the microstructure of wood. By adjusting the density and distribution of hydrogen bonds in the microstructure, the directional modification of the macroscopic properties of wood can be realized. The modification technology targeting hydrogen bonds has become an important breakthrough in the functional design of wood.

## 3. Hydrogen Bond Modification

Hydrogen bonds are widespread in many materials as intermolecular forces. When a particular chemical reagent is added, it can be combined with the hydroxyl groups of wood and other objects, forming a new hydrogen bond. The network of old hydrogen bonds can also change, and during the process, the properties of the material will change [70]. Thus, in many methods of modification, the transformation of hydrogen bonds is part of the mechanism of action. The following content will discuss in detail how changes in hydrogen bonding affect the performance of materials under various modification techniques.

### 3.1. Organic Modification

Organic modification is a modification method in which organic groups react with polar groups on the surface (such as hydroxyl groups) to form new bonds (covalent bonds/hydrogen bonds) and destroy old bonds. This process changes the polarity of the surface and adjusts the density and type of bonds (for example, from free hydroxyl bonds to esterified hydroxyl bonds), thereby improving the hydrophilicity, strength, and stability of the material. The reconstruction of the hydrogen bond network is the key to regulating the properties of materials. Organic modification includes resin impregnation, furfuryl alcohol modification, and acetylation modification, all of which focus on ‘breaking–reconstructing’ hydrogen bonds to create functional interfaces.

#### 3.1.1. Resin Modification

Wood is a porous polymer material, and thermoreactive resins are often used for its organic modification, including urea formaldehyde (UF), phenol formaldehyde (PF), and melamine formaldehyde (MF), for wood impregnation and modification (Table 1). The molecular weight of the resin directly determines its ability to penetrate the cell wall and wood cavities [71]. The known level of technique made it possible to improve the basic condition of the wood itself—permeability [72]. With the action of the urea solution, this problem can be solved. The solution dissolves the components of lignin and hemicellulose due to a weak alkaline reaction, destroying the water bonds that have formed between them. At the microscopic level, breaking hydrogen bonds leads to an increase in structural pores and softening of the cell wall, which has both high water absorption characteristics and high permeability [73,74].

When choosing a modifier for wood modification, it is necessary to consider whether it can form a hydrogen bond with wood components, thereby increasing the effectiveness of the modifier’s penetration into the wood. UF resin has this ability. Under certain conditions, urea–formaldehyde resins undergo polycondensation and cross-linking reactions. During curing, certain functional groups (-NH_2_, -NH-, -CHOHOH) can form hydrogen bonds with polar groups (-OH, -CO-, -COOH) in pine wood, thus forming an insoluble mesh structure that binds hydrogen bonds. It can effectively improve the water resistance and tensile strength of pine wood [75]. Like urea–formaldehyde resin, melamine resin is also a kind of amino resin, and after penetrating into the wood structure through diffusion, it can also undergo a polymerization reaction, which is tightly bonded to the wood fibers and forms a good hydrogen bond. It not only fills the micropores and defects within the cell wall but also forms a polymer network that disperses and resists stress under external forces. This greatly improves the hardness, abrasion resistance, corrosion resistance and deformation resistance of wood [76,77]. However, melamine resins are more chemically active compared to urea–formaldehyde resins. The fracture modulus of wood modified with melamine resin can be increased by as much as 17%, while that of wood modified with urea–formaldehyde resin is only increased by 5% [71].

**Table 1:** Classification, performance characteristics, and synthesis processes of resin adhesives.

Classification	Performance Characteristics	Raw Materials and Synthesis Conditions
Ultrasonic Frequency (UF)	Poisonous [78]; low cost [79]; curing conditions are mild [79]; fast curing speed; pH = 8.5–10.5.	Urea, formaldehyde; alkaline environment; temperature of 95 °C [80,81].
Phenol Formaldehyde Resin (PF)	Poisonous; lower cost; wide curing temperature; neutral or weakly alkaline; good electrical insulation, flame retardancy and chemical stability [82]; high char yield [83].	Phenol, formaldehyde; acid method synthesis [84,85,86,87].
Melamine-Formaldehyde Resin (MF)	High porosity and adsorption capacity [88]; cost is high; curing temperature is higher; slow curing speed; poor toughness, brittleness is high [89]; good heat resistance and scratch resistance [90].	Melamine, formaldehyde; temperature of 80 °C; spray-drying process [91].

**Figure 7:**
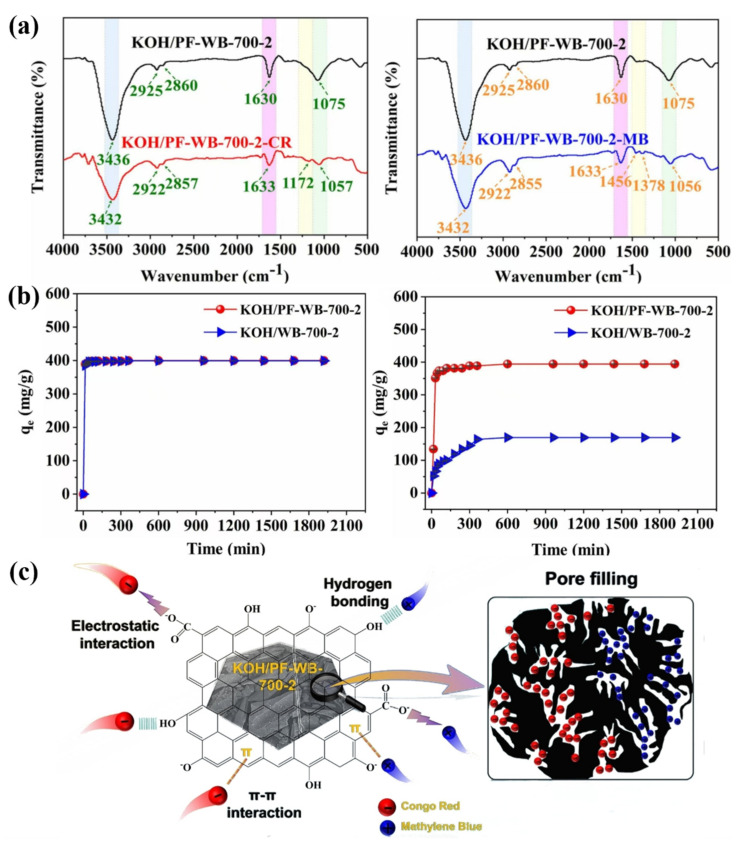
(**a**) FTIR spectra of KOH/PF-WB-700-2 before and after adsorption of Congo red (CR) and methylene blue (MB). (**b**) Rate of adsorption of MB and CR dyes by KOH/PF-WB-700-2. (**c**) Mechanism of adsorption by KOH/PF-WB biochar [94].

Resin-modified wood can be used as a raw material for the preparation of new materials due to its altered property structure [92,93]. Zhang prepared KOH/PF-WB biochar by pyrolysis and KOH activation using phenolic resin-modified southern pine wood as a carbon source. The FTIR spectra of KOH/PF-WB-700-2 before and after the adsorption of Congo red (CR) and methylene blue (MB) (Figure 7a) showed that after the adsorption of CR and MB, the characteristic peaks of -OH at 3436 cm^−1^ and C-O at 1075 cm^−1^ on KOH/PF-WB-700-2 were shifted and the C-O peak at 1075 cm^−1^ moved to 3432 cm^−1^, 1057 cm^−1^, and 1056 cm^−1^, respectively, and the intensity of the peaks decreased significantly. Hydrogen bonds can be formed between the oxygen-containing functional groups (e.g., hydroxyl and carboxyl groups) on the surface of biochar and the hydrogen atoms or electronegative atoms (e.g., nitrogen and oxygen) in the dye molecule. In addition, due to the strong directionality and saturation of hydrogen bonding, the interaction force between the dye molecules and the surface of biochar can be enhanced, thus increasing the adsorption strength. In addition, the relatively fast formation of hydrogen bonds can facilitate the contact and adsorption of dye molecules on the surface of the biochar in a short period of time. The adsorption rate of KOH/PF-WB-700-2 on MB and CR dyes increased rapidly at the early stage of adsorption (Figure 7b). In addition to the adsorption of dye molecules through the formation of hydrogen bonds, various oxygen-containing functional groups and aromatic ring structures on the surface of this biochar could also adsorb dye molecules through the combined effects of electrostatic and π-π interactions (Figure 7c). On this basis, a simple, low-cost, and highly efficient biochar adsorbent was developed [94].

In recent years, the use of resins for wood modification is no longer limited to a single type, such as UF, PF, or MF. Instead, several raw materials have been selected to synthesize new resins, which usually have more outstanding properties and improve the dimensional stability of the wood [95]. Some scholars have also suggested that the formation of hydrogen bonds does not necessarily play a good role. It is also possible that hydrogen bonds are formed between resin molecules, thus limiting the movement of the resin molecules, reducing the reactivity within the resin molecules, and affecting the rate of the curing reaction and the enthalpy of the curing reaction of the resin, which further affects the physical and mechanical properties of the resin-impregnated wood as well as the efficiency and quality of the drying process [31]. Instead of phenolic resin (PF), cashew phenol and rosin derivatives can be developed to enhance the hydrogen bond network with wood components and reduce formaldehyde release by regulating the density of phenolic hydroxyl groups.

#### 3.1.2. Furfuryolation Modification

Furfural is a commonly used wood modification technique. After the furfuryl alcohol monomer polymerizes and impregnates the wood cell wall for catalytic treatment, its main role is to increase the dimensional stability of wood [96], durability [97], hardness, modulus of elasticity [98], and flexural strength [99,100]. This method leads to a constant expansion of the wood cell wall and improves its plasticity and fixing effect during the compaction process. At the same time, lignin treatment increases the number of hydroxyl groups on the surface and significantly increases porosity, which leads to a more drastic change in the moisture content of the wood and the radial size. After furfural treatment, the gap in the wood can be effectively sealed by filling it with furfural resin, and the associated negative effects can be reduced [101].

There are differences in the ways of modifying furfurol and formaldehyde resins, which, among other things, manifest in small amounts of free formaldehyde excreted and minor environmental impacts. In recent years, furfural has been widely used in the field of wood fiber processing from biomass and is hydrated to obtain furfile spirit (FA) [102,103]. In addition, the research and development of new high-performance catalysts is becoming a reality. The furfuryl alcohol intermediate interacts with the modifier by forming hydrogen bonds on the surface, which increases the stability of the intermediate on the catalyst. It has a significant impact on the catalytic process of LCD ring hydrogenation and improves the selectivity and activity of THFK. The relationship between hydrogen bond regulation during the hydrogenation of the furfuryl alcohol ring indicates that the hydrogen bond donor group introduced by the modifier can effectively adjust the surface properties of the catalyst [104].

However, if the components of the cell walls of wood are added to the wood matrix, the fragility of the material increases, and at the same time, the toughness and bending strength can be reduced, which can lead to a significant increase in the propensity of the material to break. Recent studies have used plasticizers to ameliorate this situation, such as polyvinyl alcohol [105] and epoxidized vegetable oils [106], both of which enhance the toughness of wood by reducing the cross-link density. In a ring-opening reaction of the side chain between the plasticizer and the FA, the oxygen atom in the epoxy or ester group may form a hydrogen bond with a hydroxyl or other polar group in the FA molecule. If hydrogen bonds can be formed, it is hypothesized theoretically that the cohesive energy of the material can be enhanced, and the flexibility and mobility of the molecular chain can be dynamically regulated, thus improving the toughness and strength of the material. Unfortunately, the existence of hydrogen bonds and their related effects were not detected by characterization methods such as Fourier transform infrared spectroscopy (FTIR) or nuclear magnetic resonance (NMR).

In recent years, the utilization of organic chemicals from bio-refining and their by-products to improve the properties of wood has received extensive attention. Hu Minsu, as a by-product of bio-refining, demonstrates great potential in enhancing wood properties, especially in improving the wood’s resistance to light degradation [107]. Under heating conditions, heat-induced polymerization of humin occurs in wood. Functional groups such as furan ring, the aldehyde group, ketone group, and hydroxyl group form a cross-linked structure with wood cell wall components [108]. Hu Minsu has many similarities with polyfurfuryl alcohol, and therefore can be used as a substitute for furfuryl alcohol in wood modification. Wood treated with Hu Minsu is comparable to furfuryl alcoholized wood in terms of the modulus of elasticity, dimensional stability, and hydrophobicity, but it has better fire and photolysis resistance [109]. In addition, Hu Minsu has additional environmental and economic advantages as a by-product of the biomass conversion process.

#### 3.1.3. Acetylation Modification

The acetylation of wood is another important treatment for modified wood. Conventional acetylation requires high time and solvent requirements. Zhao et al. developed a simple impregnation method to acetylate the wood surface in a few minutes. In addition, the modified cellulose significantly lowered moisture regain and good filtration and biodegradability, which can be used in applications such as cigarette filters [110]. In the hygroscopic range of relative humidity from 0 to 98%, wood absorbs moisture mainly through hydrogen bonding within the cell walls. The hysteresis of hygroscopicity is also partly attributed to the formation and breakage of hydrogen bonds within the cell walls of wood [111]. As a result, precise acetylation can be achieved in different regions of the wood so that the hydroxyl groups in the wood cell wall are replaced by acetyl groups. Reducing the number of hydroxyl groups accessible in the wood cell wall, thus affecting the formation of hydrogen bonds, could improve the fungal decomposition performance of wood in moist and high-risk environments [112]. However, studies have shown that when the fungus adapts to an environment where the humidity of the tree’s cell wall is reduced and exceeds it, the rate of decomposition becomes the same as that of unmodified wood [113]. A decrease in the content of lignin in wood leads to a decrease in intermolecular interaction, which makes acetylated wood softer and more expensive. Once hot-pressing sealing is achieved, the modulus of rupture (Mor) and the modulus of elasticity (Moe) can be significantly improved [114].

**Figure 8:**
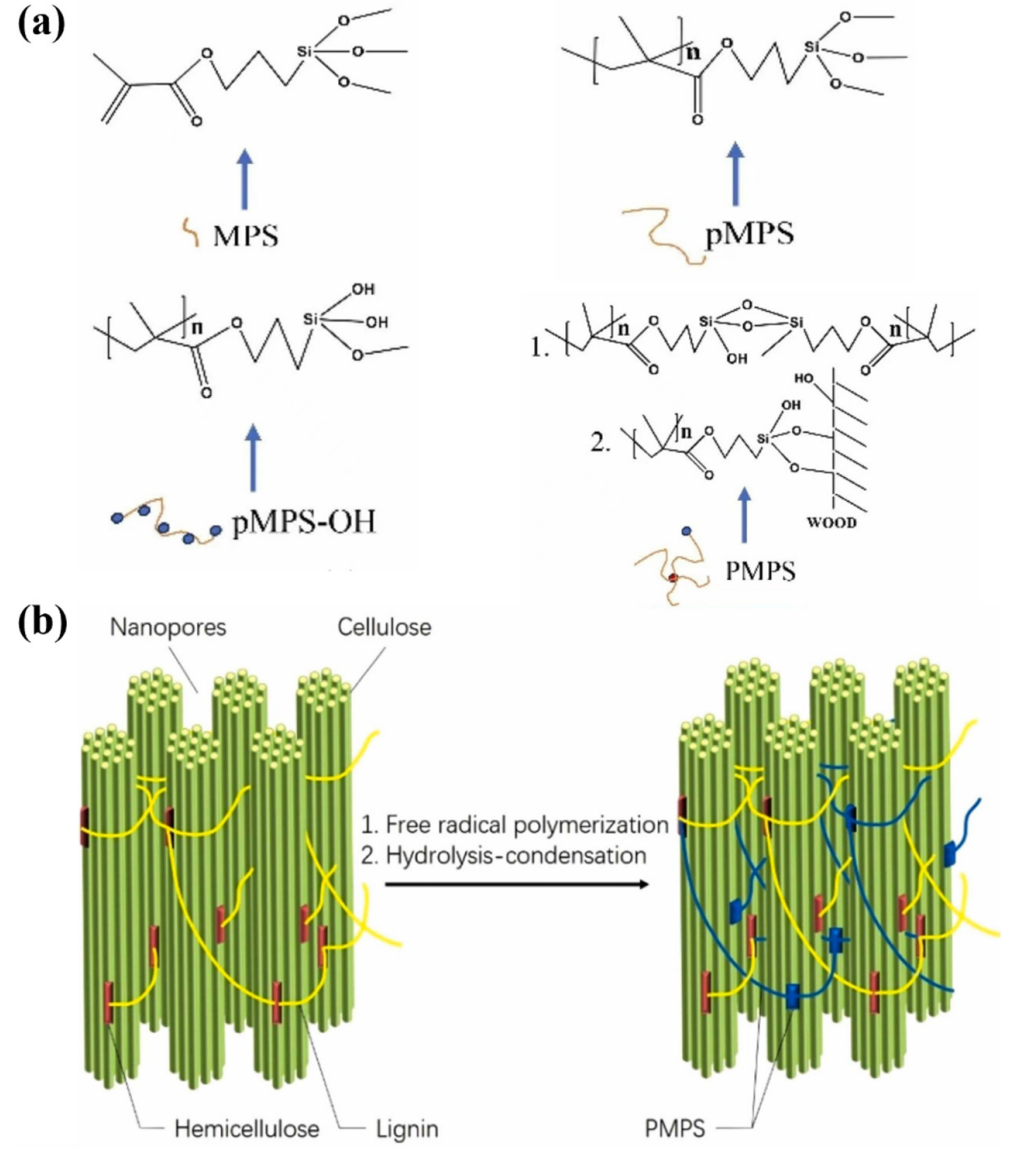
(**a**) In situ polymerization–hydrolysis–condensation reaction of γ-methacryloyloxyethyltrimethylsilane (MPS). (**b**) The generated polymer acts as a binder between the microfilaments [115].

Traditional methods of wood modification can lead to the breaking of hydrogen bonds between molecular cellulose chains, which play a key role in maintaining and improving the physical properties of wood. Based on this, researchers are making every effort to explore new wood modification techniques that do not destroy the original structure of hydrogen bonds. Qiu et al. used γ-methacrylloyloxyethyltrimethylsilane (MP) to carry out polymerization, hydrolysis, and in situ polycondensation reactions with polymer formation and Si-O-C bonding with the wood component (Figure 8a), which together acted as a binder between the microfilaments rather than disrupting the original hydrogen bonding structure (Figure 8b). This not only provides an additional bonding force but also preserves the hydrogen bonding between the chains of cellulose molecules, thus maintaining the mechanical properties of the wood [115].

Similarly, phosphorylation—a chemical modification method by the esterification of the phosphate group with the hydroxyl group in the wood component—provides a feasible path for the preparation of flame-retardant wood. This process reduces the hydrogen bond density and introduces new functional groups, such as P=O and P-OH, by replacing hydrophilic-OH with phosphoric acid groups. These new hydrogen bonds, such as P=O···H-N and P-OH···O=C, can be formed during the combustion process, thereby enhancing the stability of the char layer. The synergistic effect of covalent P-O-C bonds and the reconstructed hydrogen bond network: phosphoric acid catalyzes the dehydration of cellulose to form a heat-insulating carbon layer, and the strengthened hydrogen bonds inside the carbon layer enhance its structural integrity. Studies have shown that in the case of a small amount of load, the limiting oxygen index (LOI) of phosphorylated wood is >25%, the heat release rate is also greatly reduced, and the mechanical properties are almost unaffected. This ‘covalent hydrogen bond’ dual regulation mechanism makes phosphorylation an effective hydrogen bond engineering strategy for achieving flame-retardant functional wood [116,117].

Three organic modification techniques—resin, furfuryolation, and acetylation—all focus on regulating hydrogen bonding networks. Resin modification improves wood waterproofing and strength via hydrogen bonds and covalent cross-linking, also expanding uses like biochar adsorption. Furfuryolation enhances dimensional stability by filling pores with polymers, though plasticizer effects need clarification. Acetylation replaces hydroxyl groups with acetyl groups to reduce hydrogen bonding and boost decay resistance but lowers hardness and costs more. These methods optimize properties by breaking, recombining, or substituting hydrogen bonds. Future research should prioritize eco-friendly modifiers, strengthen hydrogen bond synergy with other interactions, and explore ways to preserve wood’s original hydrogen bonding for sustainable composites.

### 3.2. Inorganic Modification

The inorganic modification of hydrogen bonds plays a key role through the interaction between inorganic particles and wood polar groups. Inorganic materials such as silica and calcium carbonate can enhance the interfacial bonding of wood, adjust viscoelastic behavior, and improve properties such as water resistance and flame retardance by forming hydrogen bonds or altering the existing hydrogen bond network. The following will analyze the function of hydrogen bonds in the interaction between inorganic particles and the wood matrix from typical modification techniques.

#### 3.2.1. SiO_2_ Modification

Silicon dioxide impregnation is an effective wood-processing technique. SiO_2_ particles form strong hydrogen bonds with hydroxyl groups in wood components, which enhances the interfacial bonding between the nanoparticles and the wood matrix, facilitates stress transfer between the particles and the wood matrix, and improves the wood’s ability to absorb energy and resist the expansion of cracks. Moreover, under humid conditions, water molecules further promote the aggregation of SiO_2_ nanoparticles and their hydrogen bonding with wood constituents under humid conditions, thus increasing the impact strength of wood [118].

By adding a simple environmental requirement to the silica impregnation operation—negative vacuum pressure—silica nanoparticles can be uniformly distributed in the wood’s vascular bundle system, effectively blocking the pores. At the atmospheric pressure of −90 KPa, silica nanoparticles showed a more excellent enhancement mechanism, which was manifested in the significant increase in sample density and the decrease in water absorption capacity, which was 7.32 times lower than that of the initial sample [119].

#### 3.2.2. CaCO_3_ Modification

As an inorganic mineral, calcium carbonate can also change the original hydrogen bond network structure of wood. The deposition of calcium carbonate in wood can be well achieved through the exchange and circulation of chemical solutions. While increasing the strength, calcium carbonate has the potential to fix carbon, which can be used to reduce the thermal degradation rate of wood and increase the carbon residue rate. In addition, further hot pressing can reduce the gap between the particles and the matrix, thereby enhancing the viscoelasticity of the wood. It is worth noting that the dynamic load of the hydrogen bond network regulates the viscoelastic behavior, which affects the flexibility of the wood molecular chain, the interaction between the chains, and the energy dissipation mechanism, thereby affecting the energy storage modulus and loss modulus of the wood [120,121].

With the help of bionic enzyme mineralization technology, urease is anchored in the cell wall of the wood by catalyzing the hydrolysis of urea, and the resulting bicarbonate ions can react with calcium ions, forming calcium deposits. Poly-L-aspartic acid is used as a supplement that promotes the heterogeneous formation of embryos and the deposition of calcium in the cavities of wood cells. Although this is not mentioned in the paper, based on the chemical properties of the components of the cell wall of wood, it can be hypothesized that hydrogen bonds may contribute to the adsorption and deposition of a mineral precursor on the surface of the cell wall. When mesocrystalline CaCO_3_ is introduced, hydrogen bonding also improves its compatibility with the cell wall at the phase interface, which contributes to the formation of a more stable overall structure. When the mineralization process takes place again, more CaCO_3_ continues to accumulate outside the cell wall, leading to a significant increase in the amount of sediment. After a series of changes, the surface hardness of this type of mineralized wood increased significantly. Hardness increased by about 38% in the cross-section and more than 61% in the longitudinal direction. At the same time, tests showed that the fire resistance of this material during the application of flame retardants significantly improved. For example, the oxygen limit index increased from 19.8% at baseline to 24.4%, indicating better protection against combustion [32]. It is worth noting that although CaCO_3_ mineralization improves the flame retardancy, the hydrogen bond-mediated interface is prone to microcracks in the long-term water immersion–drying cycle, which weakens the toughening effect. The hydrophobic coating should be combined to reduce the water-induced damage of the hydrogen bond network.

**Figure 9:**
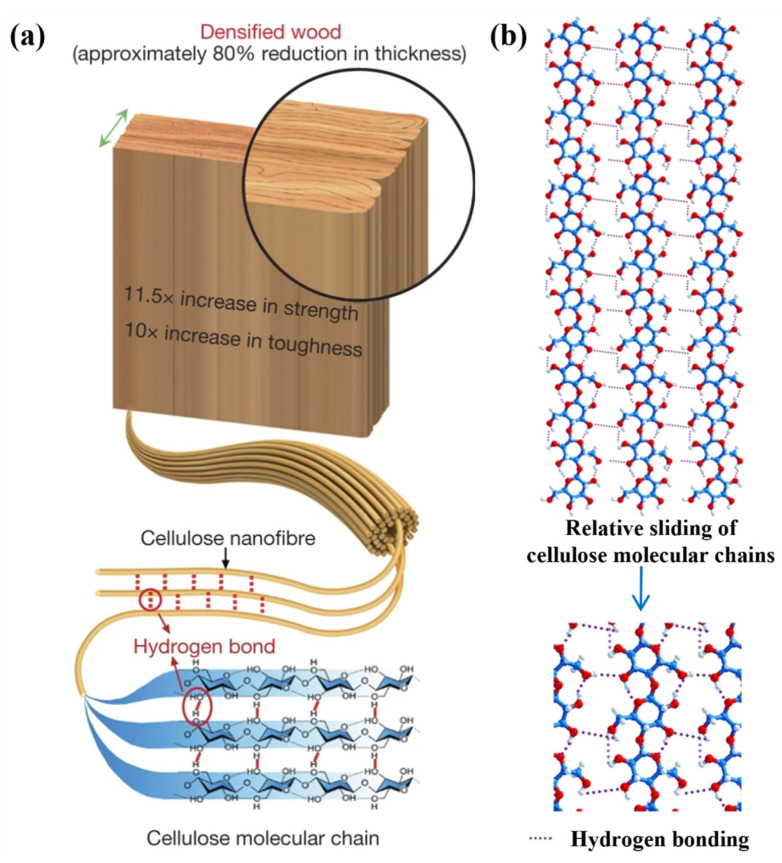
(**a**) Cellulose nanofibers are highly aligned and adjacent fibers form hydrogen bonds. (**b**) Relative sliding between densely packed wood cell walls involves repetitive processes of formation, breaking, and re-formation of a large number of hydrogen bonds on a molecular scale [123].

In wood modification, hydrogen bonds have different functions. This is reflected not only at the molecular level, but it also affects the physical properties of the wood, such as the hardness and modulus of elasticity. By changing the internal structure of hydrogen bonds, chemical modification can change the moisture absorption properties and other properties of wood. The breakdown of components in the preparation of charcoal can be accompanied by the destruction of hydrogen bonds, which reflects the excretion of hydrogen-containing substances from wood, such as moisture. It is an effective way to prove the mechanism of action of coal and the properties of modified wood [122]. After boiling in an aqueous solution containing NaOH and Na_2_SO_3_, some of the lignin and hemicellulose are removed, and then the cell wall of the wood is completely decomposed and compressed by hot pressing, which can increase the strength of the wood. During this process, hydrogen bonds are formed between many neighboring fibers (Figure 9a), which is caused by the highly oriented mood of cellulose nanovolocons. Relative sliding between the cell walls of tightly adhering wood involves the process of repeatedly forming, breaking, and reshaping a large number of hydrogen bonds at the molecular level (Figure 9b), which causes the entire tree to consume more energy than logs. This mechanism increases crack resistance and at the same time significantly increases slip resistance between adjacent fibers, which ultimately increases the strength and viscosity of the whole tree [123].

### 3.3. Organic–Inorganic Synergistic Modification

With the development of modernization, materials that have undergone only organic modification or inorganic modification alone have gradually failed to meet the industrial demands. In order to realize the multifunctionalization of the material, the material properties obtained by the combination of the two are better [124]. For instance, inorganic modification can enhance the inherent properties of wood, while organic modification can impart special functions to the wood. This process enables the production of intelligent wood with excellent mechanical properties. During these modification processes, hydrogen bonds often play a significant role.

Thermochromic wood, as a new type of intelligent material, can sense temperature changes and store thermal energy. It can be composed of a phase-change energy storage complex (TPCM) formed by silica particles encapsulated in lauroyl tetracarboxylate and red methyl. Silica combines with the hydroxyl chemical structure of cellulose and lignin through hydrogen bonds, which effectively enhances the strength of the internal structure of the wood matrix and further enhances the overall strength and hardness of the wood. While improving the strength of wood, it makes wood an excellent temperature-sensitive and color-changing phase-change material [125].

In the manufacturing of high-strength superhydrophobic wood, hydrogen bonds promote certain chemical reactions during a one-step process. For example, after treating the wood surface with sodium hydroxide (NaOH), the number of hydroxyl groups on the wood surface will increase. These hydroxyl groups use hydrogen bonds to accelerate the hydrolysis and condensation reaction of subsequent silane binders (such as ethylene triethoxysilane, VTES). The result is a stable chemical compound. Bonds are formed on the wood surface, which increase the mechanical stability and durability of the coating [126]. Research is also underway to optimize the superhydrophobic properties of wood. It is noted that SiO_2_ nanoparticles rich in hydroxyl groups (-OH) can form hydrogen bonds with hydroxyl groups of cellulose and hemicellulose in wood, which contributes to the attachment of SiO_2_ nanoparticles and their uniform distribution over the surface of wood. In this study, a polymethyl hydrogen siloxane (PMHS) chain was used as a binder between SiO_2_ nanoparticles, and the formation of hydrogen bonds can also enhance the binding effect. The contact angle of wood with water modified with SiO_2_/PMHS was significantly increased, and the rate of water absorption was significantly reduced, which provides an important impetus to the realization of the superhydrophobic and self-cleaning properties of wood [33].

The sol–gel process allows the application of hybrid inorganic–organic films to various substrates at room temperature, including wood, which allows the surface modification of various materials [127,128]. The use of hybrid organic–inorganic materials as a protective coating of wood increases water resistance and corrosion resistance, and the mechanical properties of the wood will be greatly improved [129]. This coating can effectively block the penetration of water and harmful substances, while maintaining the strength and stability of wood. When preparing the organic–inorganic hybrid polymer, monomers containing hydroxyl groups (such as glycidyl methacrylate, GMA) and cross-linking agents were used. During the polymerization process, these monomers may form hydrogen bonds with the hydroxyl groups in the wood, which helps the polymer to effectively fill and fix in the wood pores. The siloxane groups of the coupling agent may react with the hydroxyl groups on the wood surface, while its methacryloyloxy groups participate in polymerization. In this process, hydrogen bonds may also promote the interaction between the coupling agent and wood. The coating is evenly distributed on the wood surface and has good adhesion. In conditions of non-hydrolysis, the presence of hydrogen bonds can protect the Si-O-Zr bond from water degradation and cause degradation by preventing the occurrence of phase separation in mixed materials, which is very important for maintaining the permeability and hardness of the coating [34,130]. In the process of using silica/polyethylene glycol (SiO_2_/PEG) composites to modify wood, it is necessary to use the sol–gel process to create a silica network structure. The physical interaction between this structure and hydrogen bonds and other polyethylene glycol (PEG) components can not only prevent PEG leakage during the phase transition process but also delay the moisture absorption process of wood, which helps to improve the overall stability and durability of energy storage wood [131].

The hydrogen bond regulation mechanism runs through the organic, inorganic, and synergistic modification technology of wood (Table 2). Through the breaking, recombination, and synergistic effects of hydrogen bonds, the directional optimization of material properties such as water resistance, reinforcement, and flame retardancy is achieved. Organic modifications are primarily dominated by chemical bonding and hydrogen bond restructuring, while inorganic modifications rely on the enhancement of interfacial interactions through hydrogen bonds between particles and the matrix. Synergistic modifications mediate the complementary enhancement of organic and inorganic components via hydrogen bonds. Future research should further reveal the dynamic action laws of hydrogen bonds, develop biomass-based green modifiers, and construct a synergistic modification system using multiple technologies. This will promote innovative breakthroughs in the functionalization, intelligence, and sustainable utilization of wood-based composite materials, providing theoretical and technical support for the efficient conversion of biomass resources.

## 4. Synergistic Enhancement of Hydrogen Bonds and Chemical Bonds

Hydrogen bonds relate to the mechanism of interaction at the molecular level of cellulose, and the physical and chemical mechanism behind it involves many complex phenomena that have yet to be investigated [49]. Hydrogen bonds occurring simultaneously on adjacent atoms will have a synergistic reinforcement effect by secondary interactions [22]. The use of special modification methods to regulate the bond between hydrogen bonds and other chemical bonds or molecular structure functions can significantly increase the efficiency of improving the operational properties of wood. For instance, hydrogen bonds and covalent bonds enable the chitosan and tannic acid (CS@TA) nanospheres to be uniformly dispersed in the soy protein system. The formation of ionic bonds and hydrogen bonds further densifies the internal structure of the adhesive, effectively preventing water penetration. The synergistic effect of hydrogen bonds, covalent bonds, and ionic bonds results in a more compact and stable internal structure of the adhesive, thereby significantly enhancing the dry shear strength and wet shear strength of the adhesive [132]. At this stage, some researchers have made progress in this area.

**Figure 10:**
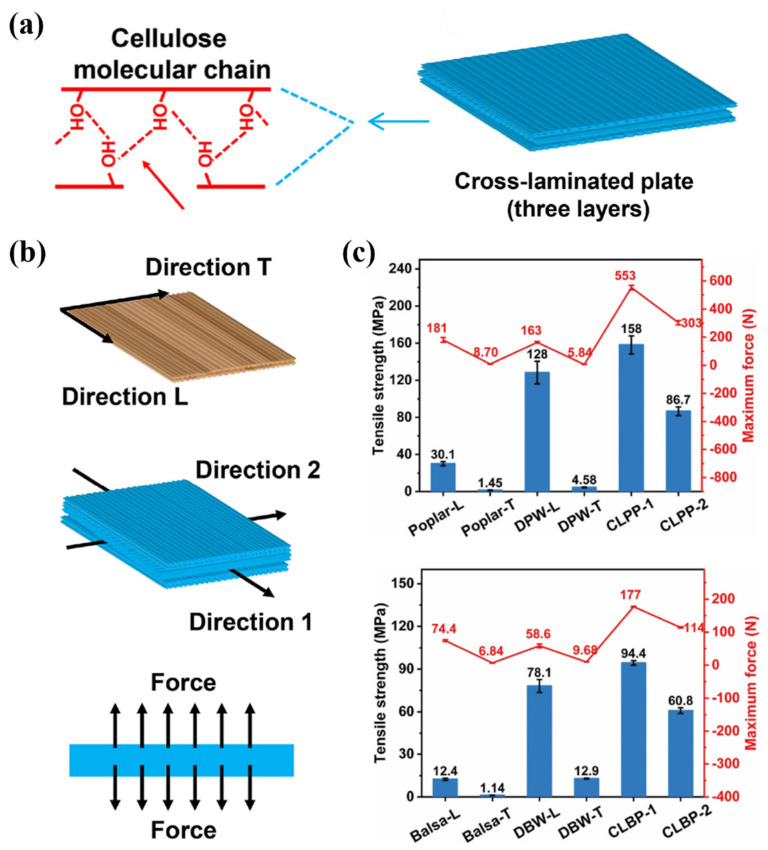
(**a**) Natural wood is cleaned, cross-compressed and densified, with each piece connected to the other by rich hydrogen bonds. (**b**) Tensile and interfacial bond strength tests of modified poplar (DPW) and balsa (DBW) along different orientations. (**c**) Tensile strength of modified poplar (DPW) and balsa (DBW) in all directions [133].

Sun et al. performed a series of operations on natural wood with lignin, including cleaning, cross-linking, pressing, and sealing, and wood shavings were connected by a large number of hydrogen bonds (Figure 10a). During the whole process, the covalent bond connection between cellulose fiber structures, the three-layer structure formed by hot pressing, and the synergistic effect of hydrogen bonds between hydroxyl groups were fully considered. Excellent mechanical properties (158 MPa) were prepared by a simple process. Subsequently, the researchers conducted tensile strength and interfacial bonding tests on modified poplar (DPV) and light wood (DBV) in multiple directions (Figure 10b), and the interfacial bonding strength was 1.13 MPa. The results showed that the tensile strength of the two tree species in all directions was higher than that of the untreated sample (Figure 10c).

**Figure 11:**
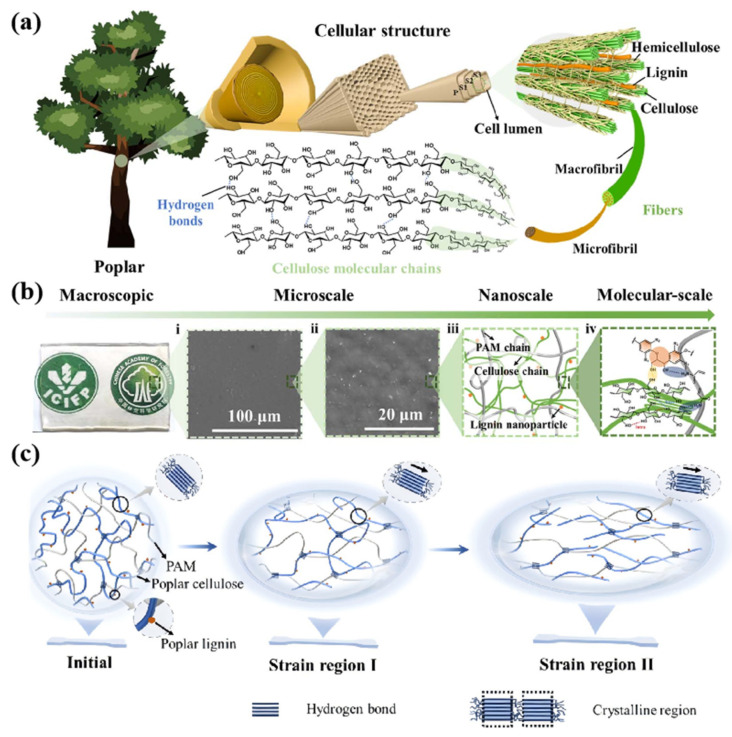
(**a**) Structure of natural wood. (**b**) Different micro-scale structures of hydrogels. (**c**) Water-induced self-assembly process forms non-covalently cross-linked cellulose and lignin nanoparticle network structures [134].

The removal of lignin further reveals the surface of the wood fibers, while the pressing treatment is combined with the introduction of water molecules for the tighter weaving of cellulose fibers. After drying, a strong network of hydrogen bonds is formed between the adjacent fibers. Throughout the process, different mechanisms influence each other and coordinate their actions, which leads to an increase in density, strength, and strength indicators [133]. Such synergistic mechanism effects have been considered not only in wood modification but also utilized in some wood biomimetic materials. Xie et al. extracted cellulose and lignin complexes from poplar and designed a hydrogel with an isotropic layered structure (Figure 11a) by mimicking the structure of natural wood (Figure 11b). Through a water-induced self-assembly process, a non-covalently cross-linked cellulose network and lignin nanoparticles were formed. In this network, lignin nanoparticles, cellulose type II crystal structures are entangled (hydroxyl groups on the cellulose molecular chains form orderly stacked crystal regions through hydrogen bonding), and non-covalent interactions occur (Figure 11c). It overcomes the challenge of achieving high strength (4.6 MPa), toughness (6.0 MJ/m^3^), electrical conductivity (8.2 mS/cm^1^), and isotropy simultaneously in conventional hydrogels [134].

Unlike the above studies that enhance wood properties from different aspects, the study by Kuai et al. focuses on the gradual enhancement of wood properties, which ultimately overcomes the swelling problem of water-absorbing thickness that is common in densified wood. Through a chain of alkaline chemical pretreatment, multi-stage cyclic impregnation, and high-temperature densification, lignin and hemicellulose were first removed to obtain highly oriented cellulose, and a large number of hydrogen bonds were generated between these celluloses, which initially enhanced the internal structure of the wood. Then, the wood was impregnated with sodium silicate solution for four cycles under positive and negative pressures to solidify with cellulose and form a Si-O-Si structure, which further improves the durability and stability of the wood; finally, the high-temperature densification treatment produces Si-O-C bonds to form a pike-shaped heterostructure, which once again enhances the hydrogen bonding and the arrangement of cellulose, making the wood internally connected more tightly. After modification, the hardness, flexural strength, elastic modulus, and compressive strength were increased by 3.9 (28.4 MPa), 6.0 (330 MPa), 3.4 (26 GPa), and 28.2 (235.1 MPa) times, respectively, and the thickness expansion rate was 1.0% after 72 h of water absorption [135].

**Figure 12:**
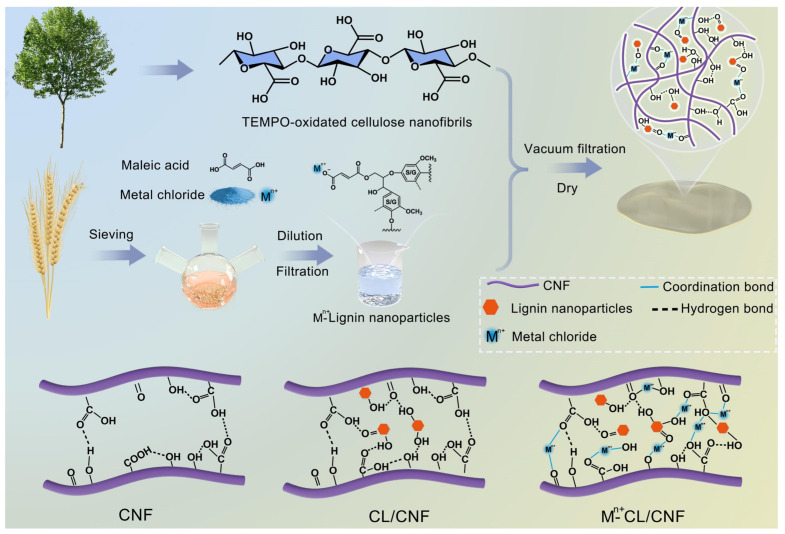
Schematic diagram for the fabrication process of metal ion and hydrogen bonding synergistically mediated carboxylated lignin/cellulose nanofibrils composite film [136].

Making full use of the synergistic effect of hydrogen bonds and other structures, while improving the performance of materials, it also fully retains the inherent advantages of wood (such as cleanliness, environmental protection, and biodegradability). Luo et al. prepared a composite film by mixing the prepared carboxy lignin with CNF, which was prepared by a vacuum filtration and drying process. The problem of lignin inhibiting the formation of hydrogen bonding between CNF was alleviated by the construction of a super-strong network between CL, CNF, and metal ions through a variety of non-covalent bonding interactions. The mechanism for enhancing the mechanical properties of the composite films was the strong hydrogen bonding formed between CL and CNF, which, combined with the electrostatic interactions between the metal ions and carboxyl groups, constructed synergistically mediated cross-linking and hydrogen bonding of the metal ions between CL and CNF. Tests show that the binding between CNF and various CLs (276–406 nN) was higher than that between pure CNF (202 nN). The composite film exhibits the highest tensile strength (180.0 MPa) and Young‘s modulus (13.0 GPa) (Figure 12) [136].

**Figure 13:**
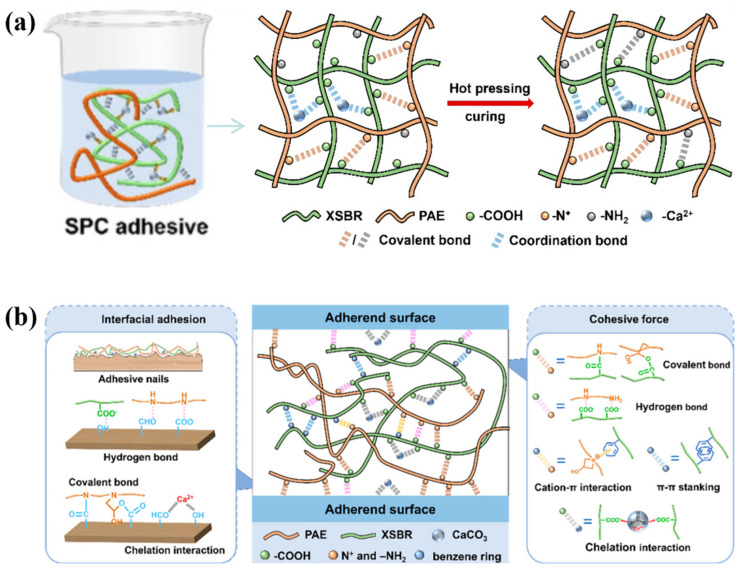
(**a**) Combination of organic and inorganic substances in the preparation of super-strong formaldehyde-free wood adhesives. (**b**) Alternative cross-linking in the modification process [137].

Beyond the aforementioned applications in the enhancement of mechanical properties of wood and biomimetic structural design, the synergistic effects of hydrogen bonding with other interaction mechanisms have also demonstrated significant value in cellulose-based environmental functional materials. For example, Zhang et al. also fully utilized the synergistic effect of hydrogen bonding with other adsorption mechanisms such as electrostatic attraction and π-π interaction (Figure 7c) [94]. The organic–inorganic constructed cross-linked network also belongs to a kind of synergistic improvement of material properties, which is mainly reflected in the interaction between organic and inorganic components. In the preparation of ultra-strong formaldehyde-free wood adhesives, organic polymers provided excellent adhesion and flexibility, while inorganic nanoparticles improved the hardness and heat resistance of the adhesives (Figure 13a). In addition, various cross-linking methods such as π-π stacking and physical cross-linking were introduced (Figure 13b) to further enhance the cohesion and stability of the adhesives. This enhanced the strength and durability of the adhesives [137].

## 5. The Future of Hydrogen Bonds and Wood Modification

**Figure 14:**
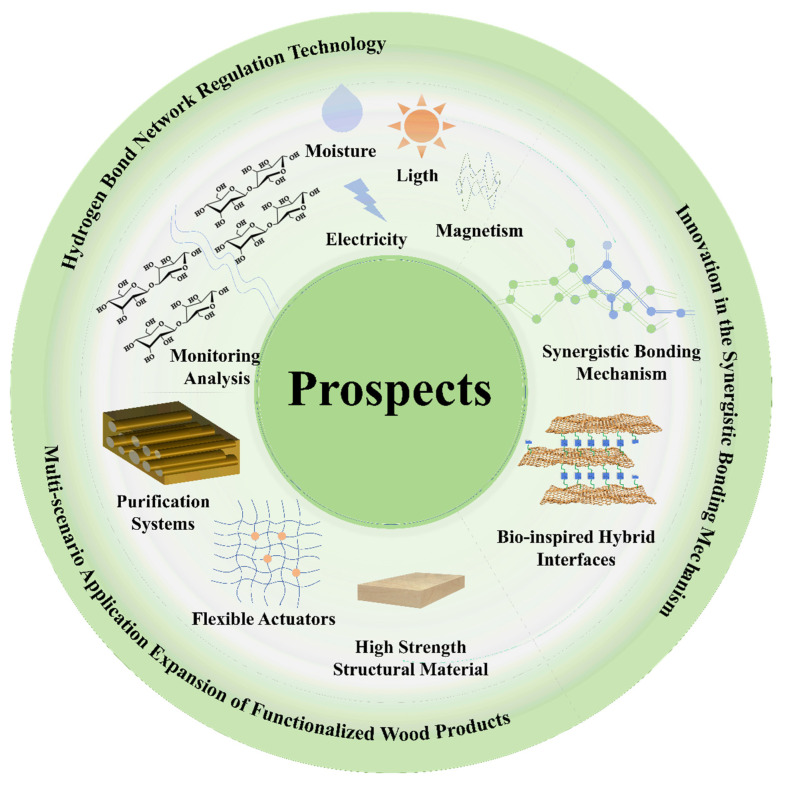
Future directions and applications of wood modification.

As a natural renewable material, the natural advantages of wood make it stand out again in the context of sustainable development. The functional modification of it to optimize its properties is the core issue to improve the utilization of wood. In recent years, the study of dynamic regulation and synergistic effect mechanisms based on hydrogen bonding networks provides a unique scientific perspective for wood modification (Figure 14). However, it is worth noting that the depth of penetration of modifying agents into wood, which is crucial for effective modification, has not been adequately addressed in the current literature. Future research should focus on quantifying the penetration depth of various modifying agents to better understand their impact on wood properties. Techniques such as confocal microscopy and depth profiling could be employed to provide detailed insights into how deeply modifying agents penetrate and interact with the wood matrix.

### 5.1. Deepening and Intelligent Design of Multi-Scale Hydrogen Bond Network Regulation Technology

Hydrogen bonding serves as the main force between the three major components of wood cell walls, cellulose, hemicellulose, and lignin, and the dynamic reorganization of the hydrogen bonding network improves the mechanical properties, hygroscopicity, dimensional stability, and other properties of the wood. Ren et al. found through molecular dynamics simulations that amorphous cellulose surfaces have high adhesion and low surface energies, which greatly improves polymer dispersion on the amorphous surfaces. They systematically discussed how crystalline and amorphous surfaces affect the interaction, adhesion properties, and diffusion behavior of polymers with wood cell wall components [138]. Moisture uptake and desorption lead to the dissociation and reconfiguration of the hydrogen bonding network, which in turn induce inter-fiber slip and changes in mechanical properties. The current technological means cannot clearly reveal the microscopic mechanisms, such as chemical bonding and intermolecular forces, between the modifier and the wood components. The design and development of new modified wood is costly but inefficient. Future research needs to further incorporate multi-scale simulation techniques (e.g., coupled quantum mechanics–molecular dynamics models) to accurately analyze the fracture energy barriers and recombination paths of hydrogen bonds under different humidity, temperature, and external field effects [139]. For example, the quantum mechanics model of the hydrogen bond fracture energy barrier of cellulose–water molecules can be constructed, and the hydrogen bond recombination path under hygrothermal conditions can be simulated by molecular dynamics. The simplified force field of wood components was developed to realize the large-scale simulation of the permeation behavior of nano-SiO_2_ in the pores of the cell wall and to optimize the particle size and dispersion. However, current molecular dynamics simulations are mostly based on idealized models, which oversimplify the competition between hydrogen bonds and hydrophobic interactions and π-π stacking in real wood.

In addition, the “programmability” of dynamic hydrogen bonding networks will be an important direction [140]. Previous studies have shown that the inflow and outflow of water in cellular structures can stimulate volume changes and elastic deformation. By constructing a complex localized force field at each node, it is possible to induce the directional arrangement of microfilaments and use the periodic breaking and reorganization of hydrogen bonds to modulate the properties of the material [141]. In the future, we will be able to focus on external influences such as light, electricity, and magnetism to study their impact on renewing hydrogen bond networks, and then develop new wooden compositional materials with intelligent properties such as self-healing and shape memory.

Hydrogen bond regulation technology has injected new vitality into the composite modification system. By precisely controlling the formation and breaking of hydrogen bonds, the internal interaction forces within the material can be optimized, thereby generating a synergistic effect with other modification techniques. This synergistic effect not only significantly improves the performance of the material but also promotes the continuous innovation and development of the composite modification system, bringing new breakthroughs to the field of materials science.

### 5.2. Innovation in the Synergistic Bonding Mechanism of Organic–Inorganic Composite Modification Systems

Traditional single-component modification often faces trade-offs between mechanical strength, toughness, and multifunctionality, which often makes it difficult to balance mechanical strength with the requirements of toughness and functionalization. An organic–inorganic composite system can use the regulation of the orientation of hydrogen bonds and the interaction between chemical bonds to create a multi-stage interface structure. These chemical bonds include covalent bonds, ionic bonds, and Van der Waals interactions. In addition, the integration of fireproof, conductive, photocatalytic, and other functional characteristics of inorganic materials themselves helps to overcome the limitations of traditional modification and conduct detailed experimental research. For instance, TiO_2_ nanoparticles with excellent photocatalytic properties are uniformly loaded on the surface or cell walls of wood. As a result, the wood not only retains its original structural characteristics but also acquires the photocatalytic function of TiO_2_ [142]. Thus, wood can be converted from a simple structural material into an intelligent multifunctional material. However, it should be noted that its potential ecological risks (such as soil microbial toxicity) and the non-degradability of modifiers are often ignored. For example, the Si-O-Si network cross-linked by a silane coupling agent is difficult to recycle after the end of the life cycle, and priority should be given to the development of biomass-based dynamic covalent substitutes in the future.

The combination of organic–inorganic modifiers is not just physical mixing. They act synergistically through chemical bonds at the molecular level, thus improving the interface and functional interaction. As an example, a Silicon–cellulose composite system can be attached, in which the amino group of the Silicon Monomer interacts with the hydroxyl group of the cellulose through a solid hydrogen bond. At the same time, a hydrolysis and condensation reaction occurs in the siloxyl group. On the surface of the wood, a three-dimensional network of Si-O-Si is formed, and the cellulose chain is fixed with covalent bonds. This double-bonding strategy involves pre-assembling hydrogen bonds and fixing covalent bonds, which significantly increases the strength of the interphase bonds and allows for a reduction in the fluidity of the modifier.

Materials can perform specific functions using dynamic networks of covalent and hydrogen bonds. Such synergistic mechanisms are effective in improving the stability of the interface, and relying on the stimulation of the external field, the state of coupling can also be modulated, making the function flexible. For example, the reversible transformation of hydrogen or covalent bonds can be achieved at a certain temperature, and then the microstructure can be used to adapt macroscopic properties, thus helping the material to adapt to various harsh environmental conditions. In the process, a new mindset developed by Smart Wood also emerged. Smart Wood herein refers to wood-based materials capable of a dynamic response to external stimuli (e.g., humidity and temperature) via programmable hydrogen bond networks, enabling functions such as self-healing or shape memory.

The excellent properties of many natural biological materials come from a multi-layered ordered structure on the border of organic and inorganic interaction that inspires wood modification. For example, the organic–inorganic gradient interface is created in the cell wall of wood by a layered impregnation method, while the bionic hybrid interface is designed to produce a composite system with a gradient density of hydrogen bonds. For example, mimicking the structure of “brick-sand” (a typical microstructure common in the construction industry), wood is modified at multiple levels by various transverse or chelate hydrogen bond modifiers, such as nanocellulose, chitosan, and titanium dioxide [143]. The molecular interaction between the surfaces of the phase interface helps to increase the bonding force on the surface of the interface, and the resulting multi-stage composite system is more effective in improving the mechanical properties of wood. If it is possible to control the physical and chemical reaction between each modifier and the molecular structure of the wood, the wood is expected to have intelligent functions. In recent years, scientific research has widely propagated the use of the structural-limiting effect of the material itself. In wood modification and related experiments, some inorganic nanoparticles, such as SiO_2_ (silicon dioxide) and MOF, can, through a spatial constraint effect, penetrate the wood nanophores to achieve the effect of increasing the density of hydrogen bonds [144].

The traditional modification of composites is often based on the use of toxic solvents or high-temperature and pressure conditions. In order to respondt to the concepts of sustainability and environmental protection, both in the selection of raw materials and in the optimization of processes, one can move to environmental modification. It is believed that obtaining inorganic precursors on a biological basis and replacing the original chemical raw material with inorganic materials derived from biomass have great opportunities for development. In addition, due to the destruction of the structure of hydrogen bonds in wood waste, a pretreatment procedure based on the use of ionic liquids or low-eutectic solvents can be developed to restore the density of hydrogen bonds between molecular cellulose chains, allowing for the restoration of the mechanical properties of old wood.

The innovation of the composite modification system is the key to enhancing the properties of wood. By introducing new modification technologies and materials, the mechanical properties, durability, and adsorption performance of wood can be significantly improved. The enhancement of wood properties also makes it applicable in more fields. For instance, high-strength and durable wood composites can be used in the construction and automotive industries; efficient adsorption performance of biochar can be utilized for wastewater treatment and environmental restoration.

### 5.3. Multi-Scenario Application Expansion of Functionalized Wood Products

With technological innovations such as hydrogen bond regulation, composite modification, and environmental modification, functional wood gradually freed itself from the limitations inherent in traditional application areas and gradually demonstrated the characteristics of high performance, intellectuality, and multifunctionality.

By using recombinant cellulose hydrogen bond networks and notechnology, the mechanical properties of functionalized wood can be significantly improved. The strength of wood with modified longitudinal tensile density outweighs the strength of some steel, so it is suitable for making light buildings, bridges, and vehicles. Wood that has been reinforced and modified can be used for building structures and is widely used in load-bearing structures and prefabricated building modules. In the field of transport, for example, in the production of high-speed rail interiors, Caracas kuzova new energy vehicles, etc., it can also be used to replace aluminum alloy, which has both light and damping properties. Although the strength of modified wood is comparable to that of metals, the decrease in toughness caused by resin impregnation or acetylation may limit its impact resistance applications. In addition, the existing research mostly ignores the irreversible degradation of the hydrogen bond network in long-term hot and humid environments, which requires reasonable engineering material selection.

By introducing a thermosensitive/hygroscopic polymer and a network of hydrogen bonds into wood, a “Smart” Wood with reversible deformation and self-healing properties is created, which opens up new possibilities for its application in the Internet of Things and robotics. For example, moisture-dependent deformed wood can automatically adjust ventilation openings based on changing environmental humidity to optimize the energy efficiency of buildings and is suitable for finishing the exterior walls of buildings [145]. In addition, a flexible drive designed on the basis of wood and hydrogel composite materials can perform capture tasks by responding to changes in humidity and temperature and can also be used in auxiliary medical equipment [146].

After MOF and nanocatalytic materials are added to the functional wood, it begins to adsorb and degrade pollutants under the action of forming the surface structure. This feature can be widely adapted to environmental recovery scenarios. Wood-based composite materials improve the conductive performance of porous structures, which allows for the more efficient adsorption of heavy metal ions and shows potential in industrial wastewater treatment and water purification. Wood modified with a photocatalyst can effectively degrade volatile organic compounds, which allows its use in indoor air purification systems and contributes to the complex photocatalytic-adsorption effect.

The structure of the material determines the direction of work. In wood, hydrogen bonds are key chemical compounds. If their structure is disturbed or reorganized, this can significantly affect the performance of the wood. Based on this relationship, it is necessary to conduct in-depth studies on the intermolecular structure of wood, focusing on this structure, studying appropriate modification techniques, or creating new modification techniques, and then working on guiding the set goal in order to obtain the ideal wood material.

## Figures and Tables

**Table 2:** Related reagents, synthesis efficiency, environmental impact, cost, mechanism of action, and performance changes in different modification methods.

Modification Methods		Main Reagents/Materials	Efficiency	Environmental Impact	Cost	Mechanism of Action	Performance Change
Organic modification	Resin impregnation	Urea formaldehyde resin (UF), phenolic resin (PF), melamine formaldehyde resin (MF)	Medium–high (mature process, curing rate varies from resin)	The formaldehyde-containing resin releases VOCs, and MF has lower toxicity but a higher cost than UF/PF	UF/PF has a lower cost and higher MF.	Resin functional groups (-NH_2_, -CH_2_OH) form hydrogen bonds with wood polar groups (-OH). After curing, a cross-linked network is formed.	Filling the cell wall defects, the waterproof, tensile strength, hardness, and other properties are improved.
	Furfurylation	Furfuryl alcohol (FA)	(Impregnation and catalytic polymerization required)	Furfuryl alcohol from biomass, less free formaldehyde	Depends on furfuryl alcohol raw materials and process costs	FA polymerization fills the pores of wood and reduces free hydroxyl groups. Hydrogen bond recombination enhances stability.	The dimensional stability, hardness, and strength are improved, but the brittleness will also increase.
	Acetylate	Acetic anhydride	The traditional method is time-consuming, and the new method such as the rapid impregnation method can be improved	Nontoxic reagent, stable product	Mainly from the cost and process of acetic anhydride	Acetyl replaces the cell wall hydroxyl group and reduces the hydrogen bond formation site.	Moisture absorption decreases, and hot pressing can further improve strength.
Inorganic modification	SiO_2_ impregnation	Silica nanoparticles	Medium (impregnation and distribution control required)	Innocuity	Depending on the cost of nanoparticles	SiO_2_ forms hydrogen bonds with wood hydroxyl groups to enhance the interface bonding. Water molecules promote hydrogen bond aggregation in humid environments.	It has high impact resistance, high density, and weak hygroscopicity.
	CaCO_3_ mineralization	Calcium carbonate	Low (requiring enzyme catalysis or solution exchange, multiple steps)	Innocuity	Depending on the reagent and process cost	Hydrogen bonds promote the adsorption of mineral precursors on the cell wall. High-temperature pressing enhances viscoelasticity.	The hardness is improved, and the flame retardancy is also improved.
Organic–inorganic synergistic modification	Organic–inorganic composites (e.g., SiO_2_/PEG and resin/SiO_2_)	Silica/lauric acid tetracarboxylate/vinyltriethoxysilane/glycidyl methacrylat, etc.	Medium-low (often involving multi-step process or sol–gel)	Depending on the specific organic/inorganic component	Depending on a variety of materials and processes	Related materials are combined with wood chemical components through hydrogen bonds and give wood different properties.	According to the relevant material changes, such as mechanical, thermochromic, superhydrophobic, and other properties.

## References

[1] Ramage M.H., Burridge H., Busse-Wicher M., Fereday G., Reynolds T., Shah D.U., Wu G., Yu L., Fleming P., Densley-Tingley D. (2017). The wood from the trees: The use of timber in construction. Renew. Sustain. Energy Rev..

[2] Ritchie H. (2021). Drivers of Deforestation. Our World in Data. https://ourworldindata.org/drivers-of-deforestation?utm_source=Rambler&utm_medium=woman&utm_campaign=transition.

[3] Hill C.A.S. (2006). Modifying the Properties of Wood. Wood Modification.

[4] Hill C.A.S. (2006). The Use of Timber in the Twenty-first Century. Wood Modification.

[5] Hill C., Hughes M., Gudsell D. (2021). Environmental Impact of Wood Modification. Coatings.

[6] Han X., Wang Z., Ding L., Chen L., Wang F., Pu J., Jiang S. (2021). Water molecule-induced hydrogen bonding between cellulose nanofibers toward highly strong and tough materials from wood aerogel. Chin. Chem. Lett..

[7] Chen C., Xu P., Wang X. (2024). Structure and mechanical properties of windmill palm fiber with different delignification treatments. J. Bioresour. Bioprod..

[8] Azar F.-Z., El Kasmi A., Lillo-Ródenas M.Á., del Carmen Román-Martínez M., Liu H. (2024). Selective biomass conversion over novel designed tandem catalyst. J. Bioresour. Bioprod..

[9] Giachi G., Capretti C., Macchioni N., Pizzo B., Donato I.D. (2010). A methodological approach in the evaluation of the efficacy of treatments for the dimensional stabilisation of waterlogged archaeological wood. J. Cult. Herit..

[10] Qiu Y., Hu C., Li J., Lai Q., Liu Z., Lin X., Zhang W. (2024). Cell Wall Modification Based on Combination Reagents to Improve Dimensional Stability of Wood with High Efficiency. J. Agric. Food Chem..

[11] Zhou Y., Zhang Y., Kan L., Wang Y., Wang K., Hu D. (2024). Aqueous modification of waterlogged archaeological wood by phenylboronic acid to reduce hygroscopicity and improve the dimensional stability. Wood Sci. Technol..

[12] Wen Y., Ji Y., Zhang S., Zhang J., Cai G. (2019). A Simple Low-Cost Method to Prepare Lignocellulose-Based Composites for Efficient Removal of Cd(II) from Wastewater. Polymers.

[13] Sun Y., Li Z., Yan Q., Zhang S. (2023). Dual hydrogen-bonding network strategy enables fabrication of robust soy protein adhesive capable of excellent bonding at ambient temperature. Ind. Crops Prod..

[14] Grabowski S.J. (2020). Triel bond and coordination of triel centres—Comparison with hydrogen bond interaction. Coord. Chem. Rev..

[15] Arunan E., Desiraju G.R., Klein R.A., Sadlej J., Scheiner S., Alkorta I., Clary D.C., Crabtree R.H., Dannenberg J.J., Hobza P. (2011). Defining the hydrogen bond: An account (IUPAC Technical Report). Pure Appl. Chem..

[16] Desiraju G.R. (2017). IUPAC definition of the hydrogen bond. Terminology and nomenclature. Found. Crystallogr..

[17] Grabowski S.J. (2024). Hydrogen bond types which do not fit accepted definitions. Chem. Commun..

[18] Arunan E., Metrangolo P., Resnati G., Scheiner S. (2024). IUPAC Recommendations: (Un)equivocal Understanding of Hydrogen and Halogen Bonds and Their (Un)equivocal Naming!. Cryst. Growth Des..

[19] Zheng S., Xu S., Wang G., Tang Q., Jiang X., Li Z., Xu Y., Wang R., Lin F. (2017). Proposed Hydrogen-Bonding Index of Donor or Acceptor Reflecting Its Intrinsic Contribution to Hydrogen-Bonding Strength. J. Chem. Inf. Model..

[20] Wu Q., Kang X., Liu Z., Liu H., Zhao X., Liu T., Wang Q. (2024). Study on the preparation of tannic acid-enhanced hydrogels and their properties. J. For. Eng..

[21] Cheng L., Wang S., Lu H., Ye J., Xu J., Wang K., Jiang J. (2024). Selective activation of CC bonds in lignin model compounds and lignin for production of value-added chemicals. J. Bioresour. Bioprod..

[22] Garcia M.R., Iribarren I., Rozas I., Trujillo C. (2023). Simultaneous Hydrogen Bonds with Different Binding Modes: The Acceptor “Rules” but the Donor “Chooses”. Chem. A Eur. J..

[23] Decato D.A., Sun J., Boller M.R., Berryman O.B. (2022). Pushing the limits of the hydrogen bond enhanced halogen bond—The case of the C–H hydrogen bond. Chem. Sci..

[24] Willems W., Altgen M., Rautkari L. (2020). A molecular model for reversible and irreversible hygroscopicity changes by thermal wood modification. Holzforschung.

[25] Olsén P., Herrera N., Berglund L.A. (2020). Polymer Grafting Inside Wood Cellulose Fibers by Improved Hydroxyl Accessibility from Fiber Swelling. Biomacromolecules.

[26] Oh Y., Park S., Jung D., Oh K.K., Lee S.H. (2020). Effect of hydrogen bond donor on the choline chloride-based deep eutectic solvent-mediated extraction of lignin from pine wood. Int. J. Biol. Macromo..

[27] ÇElİK A.E., Can A. (2023). Surface characterization of wood treated with acidic deep eutectic solvents. Eur. J. Wood Wood Prod..

[28] Jarvis M.C. (2023). Hydrogen bonding and other non-covalent interactions at the surfaces of cellulose microfibrils. Cellulose.

[29] Chen R., Tang H., Dai Y., Zong W., Zhang W., He G., Wang X. (2022). Robust Bioinspired MXene–Hemicellulose Composite Films with Excellent Electrical Conductivity for Multifunctional Electrode Applications. ACS Nano.

[30] Zhou J., Fang Z., Cui J., Zhang X., Qian Y., Liu W., Yang D., Qiu X. (2021). Wood-inspired strategy to toughen transparent cellulose nanofibril films. Carbohydr. Polym..

[31] Li Z., Zhang X., Song S., Xu K., Lyu J., Li X. (2022). Curing characteristics of low molecular weight melamine-urea–formaldehyde (MUF) resin-impregnated poplar wood. Constr. Build. Mater..

[32] Fei T., Yi H.-J., Zboray R., Yan X.-Q., Song S.-S., Ren L., Guo H., Jiang Y. (2022). Bioinspired Enzymatic Mineralization Incorporates CaCO3 Mesocrystals in Wood for Surface Reinforcement and Flame-Retardancy. ACS Sustain. Chem. Eng..

[33] Zhang X., Xiao F., Feng Q., Zheng J., Chen C., Chen H., Yang W. (2020). Preparation of SiO2 nanoparticles with adjustable size for fabrication of SiO_2_/PMHS ORMOSIL superhydrophobic surface on cellulose-based substrates. Prog. Org. Coat..

[34] Dong X., Zhuo X., Wei J., Zhang G., Li Y. (2017). Wood-Based Nanocomposite Derived by in Situ Formation of Organic–Inorganic Hybrid Polymer within Wood via a Sol–Gel Method. ACS Appl. Mater. Interfaces.

[35] Klemm D., Heublein B., Fink H.P., Bohn A. (2005). Cellulose: Fascinating biopolymer and sustainable raw material. Angew. Chem. Int. Ed..

[36] Mülhaupt R. (1999). Hermann Staudinger und die Entwicklung der Makromolekularen Chemie. Nach. Chem. Tech. Lab..

[37] Kannam S.K., Oehme D.P., Doblin M.S., Gidley M.J., Bacic A., Downton M.T. (2017). Hydrogen bonds and twist in cellulose microfibrils. Carbohydr. Polym..

[38] Zhang Y., Deng W., Wang Z., Wu M., Liu C., Yu G., Li Q., Xu C., Li B. (2025). A Green Cellulose Dissolution System for Producing Tunable Regenerated Nanocellulose Formate. ACS Nano.

[39] Xue Y., Li W., Yang G., Lin Z., Qi L., Zhu P., Yu J., Chen J. (2022). Strength Enhancement of Regenerated Cellulose Fibers by Adjustment of Hydrogen Bond Distribution in Ionic Liquid. Polymers.

[40] Zhong Y., Wang H., Hu H., Li C., Liu Z., Shen Y., Zhou Y., Huang W. (2025). Molecular-level insights into ion-regulated hydrogen bond networks: Activating cellulose crystalline domains for enhanced solar-driven water interfacial evaporation. Chem. Eng. J..

[41] Chen Q., Chen Y., Wu C. (2023). Probing the evolutionary mechanism of the hydrogen bond network of cellulose nanofibrils using three DESs. Int. J. Biol. Macromol..

[42] Shi Z., Li Y., Duan H., Wang Y., Zhang X., Cao D., Wang S., Yan X. (2025). Interfacial hydrogen bonding reorganization-assisted aqueous assembly of hydroxypropyl cellulose for robust construction of hollow nanocapsules. Int. J. Biol. Macromol..

[43] Zhang C., Keten S., Derome D., Carmeliet J. (2021). Hydrogen bonds dominated frictional stick-slip of cellulose nanocrystals. Carbohydr. Polym..

[44] Han X., Ye Y., Lam F., Pu J., Jiang F. (2019). Hydrogen-bonding-induced assembly of aligned cellulose nanofibers into ultrastrong and tough bulk materials. J. Mater. Chem. A.

[45] Peng J., Fu R., Huang Y., Lu J., Xie X., Xue Z., Chen M., Wu X., Yue H., Mai H. (2025). Influence and mechanism of NaOH concentration on the dissolution of cellulose and extraction of CNF in alkaline solvents at 15 °C. Carbohydr. Polym..

[46] Yu Y., Zhang Y., Xi L., Zhao Z., Huo S., Huang G., Fang Z., Song P. (2022). Interface nanoengineering of a core-shell structured biobased fire retardant for fire-retarding polylactide with enhanced toughness and UV protection. J. Clean. Prod..

[47] Cao Y., Chen X., Li Y., Wang Y., Yu H., Li Z., Zhou Y. (2023). Regulating and Controlling the Microstructure of Nanocellulose Aerogels by Varying the Intensity of Hydrogen Bonds. ACS Sustain. Chem. Eng..

[48] Jin Z., Chen L., Liu X., Xia R., Li W., Wang G., Zhang Q. (2025). Zeolite firmly anchored regenerated cellulose aerogel for efficient and biosafe hemostasis. Int. J. Biol. Macromol..

[49] Wohlert M., Benselfelt T., Wågberg L., Furó I., Berglund L.A., Wohlert J. (2022). Cellulose and the role of hydrogen bonds: Not in charge of everything. Cellulose.

[50] Zhang W., Qin W., Li H., Wu A.-M. (2021). Biosynthesis and Transport of Nucleotide Sugars for Plant Hemicellulose. Front. Plant Sci..

[51] Dolan G.K., Cartwright B., Bonilla M.R., Gidley M.J., Stokes J.R., Yakubov G.E. (2019). Probing adhesion between nanoscale cellulose fibres using AFM lateral force spectroscopy: The effect of hemicelluloses on hydrogen bonding. Carbohydr. Polym..

[52] Zhang L., Yu D., Chen Y., Wu C. (2024). Effect of Urea/choline chloride treatment on removing hemicellulose during alkali extraction in the preparation of high-purity dissolving pulps. Ind. Crops Prod..

[53] Khodayari A., Thielemans W., Hirn U., Van Vuure A.W., Seveno D. (2021). Cellulose-hemicellulose interactions—A nanoscale view. Carbohydr. Polym..

[54] Zhu B., Xu Y., Ge H., Wang S., Wang W., Li B., Xu H. (2023). Theoretical study of lactic acid-based deep eutectic solvents dissociation of hemicellulose with different hydrogen bonding acceptors. Ind. Crops Prod..

[55] Chibrikov V., Pieczywek P.M., Cybulska J., Zdunek A. (2023). Evaluation of elasto-plastic properties of bacterial cellulose-hemicellulose composite films. Ind. Crops Prod..

[56] Wu H., Li J., Wu Y., Gao H., Guan Y. (2021). High-Performanced Hemicellulose Based Organic-Inorganic Films with Polyethyleneimine. Polymers.

[57] Carrier M., Fournet R., Sirjean B., Amsbury S., Alfonso Y.B., Pontalier P.-Y., Bridgwater A. (2020). Fast Pyrolysis of Hemicelluloses into Short-Chain Acids: An Investigation on Concerted Mechanisms. Energy Fuels.

[58] Zhu X., Zhang C., Ma H., Lu F. (2022). Stereo-Recognition of Hydrogen Bond and Its Implications for Lignin Biomimetic Synthesis. Biomacromolecules.

[59] Kang H.J., Lee Y.J., Lee J.K., Nurika I., Suhartini S., Choe D., Kim D.H., Choi H., Murphy N.P., Kim H.Y. (2024). Production of chitosan-based composite film reinforced with lignin-rich lignocellulose nanofibers from rice husk. J. Bioresour. Bioprod..

[60] Li Z., Chen C., Xie H., Yao Y., Zhang X., Brozena A., Li J., Ding Y., Zhao X., Hong M. (2022). Sustainable high-strength macrofibres extracted from natural bamboo. Nat. Sustain..

[61] Wei X., Jihun Z. (2023). Fundamental research and application progress of transparent wood interface. J. For. Eng..

[62] Tang Q., Yuan X., Zou M., Zhang L., Chang L., Chen X., Zhang J., Zhou G., Gao K., Guo W. (2024). Mismatched Refractive Index Strategy for Fabricating Laser-Driven Wood Diffusers from Bulk Wood for Illumination Applications. Adv. Mater..

[63] Zheng M.-W., Lang Y., Han X., Jia L., Zeng H., Li C.-J. (2024). Hydrogen-bond-promoted native lignin degradation via PCET process enabled by visible light. Cell Rep. Phys. Sci..

[64] Zhu G., Shi S., Zhao L., Liu M., Gao J., Xu J. (2020). Catalytic Activation of Carbon–Hydrogen Bonds in Lignin Linkages over Strong-Base-Modified Covalent Triazine Frameworks for Lignin Oxidative Cleavage. ACS Catal..

[65] Zhang L., Zhao D., Feng M., He B., Chen X., Wei L., Zhai S.-R., An Q.-D., Sun J. (2019). Hydrogen Bond Promoted Lignin Solubilization and Electrospinning in Low Cost Protic Ionic Liquids. ACS Sustain. Chem. Eng..

[66] Mu L., Shi Y., Wang H., Zhu J. (2016). Lignin in Ethylene Glycol and Poly(ethylene glycol): Fortified Lubricants with Internal Hydrogen Bonding. ACS Sustain. Chem. Eng..

[67] Kumar N., Taylor B.R., Chourasia V., Rodriguez A., Gladden J.M., Simmons B.A., Choudhary H., Sale K.L. (2025). Multi-scale computational screening and mechanistic insights of cyclic amines as solvents for improved lignocellulosic biomass processing. Green Chem..

[68] Cao D., Gao Q., Sun H., Feng X., Zhu J., Lu X., Mu L. (2025). Decoding solvent-lignin interactions: The role of hydrogen bonding in enhancing solubility. Chem. Eng. Sci..

[69] Diao M., Wang D., Wu H., Liu L., Lipponen J., Yao J. (2024). Mechanically robust, waterproof, fast curing lignin-based waterborne polyurethane with hierarchical hydrogen bonding network. Ind. Crops Prod..

[70] Yu Y., Tyrikos-Ergas T., Zhu Y., Fittolani G., Bordoni V., Singhal A., Fair R.J., Grafmüller A., Seeberger P.H., Delbianco M. (2019). Systematic Hydrogen-Bond Manipulations To Establish Polysaccharide Structure–Property Correlations. Angew. Chem. Int. Ed..

[71] Xie Y., Fu Q., Wang Q., Xiao Z., Militz H. (2013). Effects of chemical modification on the mechanical properties of wood. Eur. J. Wood Wood Prod..

[72] Altgen M., Awais M., Altgen D., Klüppel A., Mäkelä M., Rautkari L. (2020). Distribution and curing reactions of melamine formaldehyde resin in cells of impregnation-modified wood. Sci. Rep..

[73] Qin Y. (2023). Improvement of Eucalyptus urophylla wood permeability via urea treatment. BioResources.

[74] Acosta A.P., de Avila Delucis R., Santos O.L., Amico S.C. (2024). A review on wood permeability: Influential factors and measurement technologies. J. Indian Acad. Wood Sci..

[75] Zhang M.X., Huang J.W., Wang N.Y. Modification of pine-wood/formaldehyde-urea resin composites using electron-beam radiation. Proceedings of the International Conference on Mechanical Engineering, Civil Engineering and Material Engineering (MECEM 2013).

[76] Gindl W., Hansmann C., Gierlinger N., Schwanninger M., Hinterstoisser B., Jeronimidis G. (2004). Using a water-soluble melamine-formaldehyde resin to improve the hardness of Norway spruce wood. J. Appl. Polym. Sci..

[77] Xue X., Wang F. (2025). Effects of borides on flame retardant and decay resistance properties of resin modified wood. Constr. Build. Mater..

[78] Xu Y., Zhang Q., Lei H., Zhou X., Zhao D., Du G., Pizzi A., Xi X. (2024). A formaldehyde-free amino resin alternative to urea-formaldehyde adhesives: A bio-based oxidized glucose—Urea resin. Ind. Crops Prod..

[79] Liu M., Wang Y., Wu Y., Wan H. (2018). Hydrolysis and recycling of urea formaldehyde resin residues. J. Hazard. Mater..

[80] Liu M., Thirumalai R.V.K.G., Wu Y., Wan H. (2017). Characterization of the crystalline regions of cured urea formaldehyde resin. RSC Adv..

[81] Wei A., Ou M., Wang S., Zou Y., Xiang C., Xu F., Sun L. (2024). Preparation of a Highly Flame-Retardant Urea–Formaldehyde Resin and Flame Retardance Mechanism. Polymers.

[82] Yu Z.-L., Gao Y.-C., Qin B., Ma Z.-Y., Yu S.-H. (2024). Revitalizing Traditional Phenolic Resin toward a Versatile Platform for Advanced Materials. Acc. Mater. Res..

[83] Jiang P., Wang Z., Liu H., Ma Y., Wang Y., Niu J., Pang H., Wang X. (2022). Fabrication and characterization of pyrolytic carbons from phenolic resin reinforced by SiC nanowires with chain-bead structures. Ceram. Int..

[84] Wang H., Lv R., Huang Z., Liu P., Cong P., Li T. (2016). Synthesis and Characterization of a Fluorinated Phenolic Resin/phenolic Resin Blend. J. Macromol. Sci. B.

[85] Robert T.M., Roshith K.R., Unnikrishnan V., Thomas D., Santhosh Kumar K.S., Mathew D. (2025). Linear and branched alkyl chain modification of PF resin: Synthesis, pyrolysis and ablative performance at high heat flux. Polymer.

[86] Gao Z., Lang X., Chen S., Zhao C. (2021). Mini-Review on the Synthesis of Lignin-Based Phenolic Resin. Energy Fuels.

[87] Özbay G., Ayrilmis N., Ahmad M.S. (2023). Synthesis and characterization of green phenolic resin with olive oil mill wastewater. Environ. Sci. Eur..

[88] Luo J., Yang X., Tusiime R., Chen H., Liu Y., Zhang H., Yu J. (2022). Synergistic effect of multiscale BNs/CNT and 3D melamine foam on the thermal conductive of epoxy resin. Compos. Commun..

[89] Geng X., Huang R., Zhang X., Li W. (2021). Research on long-chain alkanol etherified melamine-formaldehyde resin MicroPCMs for energy storage. Energy.

[90] Kim K.S., Choi S.Y., Kim T.W., Kang M.J. (2024). Recycling Waste Melamine-Formaldehyde Resin as a Photocatalytic Enhancer for g-C3N4 Photocatalysts and Application in Photoelectrochemical (PEC) Reactions. ACS Sustain. Chem. Eng..

[91] Zhao Y., Zhang Y., Li R., Wang Z., Lou Z., Li Y. (2020). Facile Synthesis of Ultralight and Porous Melamine-Formaldehyde (MF) Resin-Derived Magnetic Graphite-Like C_3_N_4_/Carbon Foam with Electromagnetic Wave Absorption Behavior. Crystals.

[92] Zhao L., Mao Z., Xu H., Wang B., Zhong Y., Ji B., Feng X. (2025). Preparation of epoxy resin adhesives based on high phenolic nanomodified lignin particles. Int. J. Biol. Macromol..

[93] Liang J., Hu X., Li R., Qiu Y., Zhou S., Yang S., Fan D. (2024). Enhancement of pre-pressing performance of urea formaldehyde resin adhesive. J. For. Eng..

[94] Zhang Z., Zhang M., Zhao X., Cao J. (2024). High-efficient removal and adsorption mechanism of organic dyes in wastewater by KOH-activated biochar from phenol-formaldehyde resin modified wood. Sep. Purif. Technol..

[95] Kupfernagel C., Spear M.J., Pitman A.J., Ormondroyd G.A. (2023). Wood modification with phenol urea formaldehyde (PUF) resin: The influence of wood species selection on the dimensional stability. Eur. J. Wood Wood Prod..

[96] Lande S., Westin M., Schneider M. (2004). Properties of furfurylated wood. Scand. J. For. Res..

[97] Hadi Y.S., Mulyosari D., Herliyana E.N., Pari G., Arsyad W.O.M., Abdillah I.B., Gérardin P. (2021). Furfurylation of wood from fast-growing tropical species to enhance their resistance to subterranean termite. Eur. J. Wood Wood Prod..

[98] Li W., Liu M., Wang H., Yu Y. (2020). Fabrication of highly stable and durable furfurylated wood materials. Part II: The multi-scale distribution of furfuryl alcohol (FA) resin in wood. Holzforschung.

[99] Esteves B., Nunes L., Pereira H. (2011). Properties of furfurylated wood (*Pinus pinaster*). Eur. J. Wood Wood Prod..

[100] Ehmcke G., Pilgård A., Koch G., Richter K. (2017). Topochemical analyses of furfuryl alcohol-modified radiata pine (*Pinus radiata*) by UMSP, light microscopy and SEM. Holzforschung.

[101] Yang T., Cao J., Ma E. (2019). How does delignification influence the furfurylation of wood?. Ind. Crops Prod..

[102] Tang Y., Smith R.L., Yang B., Guo H., Su Y., Qi X. (2025). Efficient hydrogenolysis of furfuryl alcohol to 1,5-pentanediol over NiLa@ZrO_2_ catalyst. Chem. Eng. J..

[103] Huang Y., Zhao P., Cui K., Chen J., Wu P., Li X. (2025). Highly selective hydrogenolysis of furfuryl alcohol to 1,5-pentanediol over an efficient Ni/PrOx catalyst. Fuel.

[104] Coan P.D., Farberow C.A., Griffin M.B., Medlin J.W. (2021). Organic Modifiers Promote Furfuryl Alcohol Ring Hydrogenation via Surface Hydrogen-Bonding Interactions. ACS Catal..

[105] Wang J., Yang T., Zhang S., Cao J. (2022). Application of polyvinyl alcohol (PVA) as a toughening agent in wood furfurylation. Holzforschung.

[106] Liu M., Wang J., Yan Q., Lyu J., Lei Y., Lyu S., Yan L. (2024). Green bio-derived epoxidized linseed-oil plasticizer improves the toughness, strength, and dimensional stability of furfuryl alcohol-modified wood. Ind. Crops Prod..

[107] de Jong E., Mascal M., Constant S., Claessen T., Tosi P., Mija A. (2025). The origin, composition, and applications of industrial humins—A review. Green Chem..

[108] Sangregorio A., Muralidhara A., Guigo N., Thygesen L.G., Marlair G., Angelici C., de Jong E., Sbirrazzuoli N. (2020). Humin based resin for wood modification and property improvement. Green Chem..

[109] Ghavidel A., Hosseinpourpia R. (2024). Photodegradation stability of huminated European pine (*Pinus sylvestris* L.) microveneers. Holzforschung.

[110] Zhao T., Xing T., Cao X., Sun S. (2025). Rapid surface acetylation of cellulosic materials at room temperature by immersion method. Carbohydr. Polym..

[111] Fredriksson M. (2019). On Wood–Water Interactions in the Over-Hygroscopic Moisture Range—Mechanisms, Methods, and Influence of Wood Modification. Forests.

[112] Digaitis R., Thybring E.E., Thygesen L.G., Fredriksson M. (2021). Targeted acetylation of wood: A tool for tuning wood-water interactions. Cellulose.

[113] Beck G., Thybring E.E., Thygesen L.G. (2018). Brown-rot fungal degradation and de-acetylation of acetylated wood. Int. J. Biol. Macromol..

[114] Guo J., Wang C., Li C., Liu Y. (2022). Effect of Acetylation on the Physical and Mechanical Performances of Mechanical Densified Spruce Wood. Forests.

[115] Qiu Y., Hu C., Li J., Lin X., Zhang W. (2023). In situ polymerization-hydrolysis-condensation of γ-methacryloxypropyltrimethoxysilane in cell wall for improved physical properties of wood. Ind. Crops Prod..

[116] Wang F., Wu N., Wang M., Deng S., Jia H. (2024). Synthesis of phenylphosphorylated microcrystal cellulose biobased flame retardants and its flame-retardant modification on PLA biomaterials. Polym. Degrad. Stab..

[117] Zhang X., Fan Q., Chen C., Hao X., Ou R., Wang Q. (2023). Mechanical and flame-retardant properties of surface-densified poplar wood impregnated by waterborne acrylic resin doped with N/P/B. J. For. Eng..

[118] Lemaire-Paul M., Foruzanmehr M.R. (2023). The study of physico-mechanical properties of SiO_2_-impregnated wood under dry and saturated conditions. Wood Sci. Technol..

[119] Lemaire-Paul M., Beuthe C.A., Riahinezhad M., Reza Foruzanmehr M. (2023). The impact of vacuum pressure on the effectiveness of SiO2 impregnation of spruce wood. Wood Sci. Technol..

[120] Choi H., Dalton L.E., Peszlen I., Pourghaz M. (2024). The impacts of CaCO3 deposition in natural wood on its viscoelastic properties. Compos. Part B Eng..

[121] Zou Y., Zhang M., Li P., Xu B., Zhang Y., Zou Y. (2024). Influence of coating technology on performance of coated wood surface. J. For. Eng..

[122] Garskaite E., Karlsson O., Stankeviciute Z., Kareiva A., Jones D., Sandberg D. (2019). Surface hardness and flammability of Na_2_SiO_3_ and nano-TiO_2_ reinforced wood composites. RSC Adv..

[123] Song J., Chen C., Zhu S., Zhu M., Dai J., Ray U., Li Y., Kuang Y., Li Y., Quispe N. (2018). Processing bulk natural wood into a high-performance structural material. Nature.

[124] Gao X., Gong R., Hu X., Gu Y., Srinivasan V., Dong X., Li Y. (2025). Highly strong, fire-retardant and water-resistant wooden structural material enabled by organic-inorganic nanohybridization In-Situ coupled with cell densification. Chem. Eng. J..

[125] Zou W., Deng J., Wang Z., Sun D., Zou N. (2024). Encapsulation of thermochromic tetradecyl myristate/methyl red composite via full poplar-based cellulose/lignin/SiO_2_ framework for preparation of thermochromic wood with thermal response and storage. Int. J. Biol. Macromol..

[126] Jia S., Chen H., Luo S., Qing Y., Deng S., Yan N., Wu Y. (2018). One-step approach to prepare superhydrophobic wood with enhanced mechanical and chemical durability: Driving of alkali. Appl. Surf. Sci..

[127] Guo X., Zhang Q., Ding X., Shen Q., Wu C., Zhang L., Yang H. (2016). Synthesis and application of several sol–gel-derived materials via sol–gel process combining with other technologies: A review. J. Sol-Gel Sci. Technol..

[128] Xu T., Zhang H., Hess D.W., Chai X., Xu K., Yang X., Xie L. (2025). A fluorine-free approach for fabricating superhydrophobic coatings on bamboo using methyltrimethoxysilane (MTMS) under alkaline conditions. Prog. Org. Coat..

[129] Zhou H., Li Z., Zhang H., Kang B., Yan D., Ye Y.N., Zhu S., Yu W., Zhu F. (2025). Modifying ethyl cellulose based coatings with PDMS and TiO2 for wood protection. Colloid Surf. A.

[130] Girardi F., Cappelletto E., Sandak J., Bochicchio G., Tessadri B., Palanti S., Feci E., Di Maggio R. (2014). Hybrid organic–inorganic materials as coatings for protecting wood. Prog. Org. Coat..

[131] Xu J., Yang T., Xu X., Guo X., Cao J. (2020). Processing solid wood into a composite phase change material for thermal energy storage by introducing silica-stabilized polyethylene glycol. Compos. Part A Appl. Sci. Manuf..

[132] Liu X., Bian Y., Zhang X., Liu Z., Weng T., Wang G., Li J., Chen H., Gao Q. (2023). Preparation of strong and mildew-resistant soybean meal adhesives with self-assembled core-shell structured nanospheres. Ind. Crops Prod..

[133] Sun H., Ren Z., Ji T., Bi H., Xu M. (2021). Mechanically strong, cost-efficiency, and sustainable fully wood-derived structural materials by micro/nanoscale design. J. Mater. Res. Technol..

[134] Xie Y., Shi X., Gao S., Lai C., Lu C., Huang Y., Zhang D., Nie S., Xu F., Chu F. (2024). Biomimicking natural wood to fabricate isotropically super-strong, tough, and transparent hydrogels for strain sensor and triboelectric nanogenerator applications. J. Mater. Chem. A.

[135] Kuai B., Xu Q., Zhan T., Lv J., Cai L., Gong M., Zhang Y. (2024). Development of super dimensional stable poplar structure with fire and mildew resistance by delignification/densification of wood with highly aligned cellulose molecules. Int. J. Biol. Macromol..

[136] Luo D., Sun G., Wang Y., Shu X., Chen J., Sun M., Liu X., Liu C., Xiao H., Xu T. (2024). Metal ion and hydrogen bonding synergistically mediated carboxylated lignin/cellulose nanofibrils composite film. Carbohydr. Polym..

[137] Xue J., Liang Z., Yan Q., Zhang S., Kang H. (2024). Fabrication of an Ultrastrong Formaldehyde-Free Wood Adhesive with an Organic–Inorganic Cross-Linking Network. Nano Lett..

[138] Ren Z., Sun H., Zhou X., Chi X., Bi H., Ji T., Xu M., Cai L. (2023). Insights from molecular dynamics simulations for interfacial effects between polylactic acid and wood cell wall constituents. Compos. Part A Appl. Sci. Manuf..

[139] Bonollo G., Trèves G., Komarov D., Mansoor S., Moroni E., Colombo G. (2025). Advancing Molecular Simulations: Merging Physical Models, Experiments, and AI to Tackle Multiscale Complexity. J. Phys. Chem. Lett..

[140] Boyken S.E., Chen Z., Groves B., Langan R.A., Oberdorfer G., Ford A., Gilmore J.M., Xu C., DiMaio F., Pereira J.H. (2016). De novo design of protein homo-oligomers with modular hydrogen-bond network–mediated specificity. Science.

[141] Zhang T., Zhang D., Chen W., Chen Y., Yang K., Yang P., Quan Q., Li Z., Zhou K., Chen M. (2023). Shape and Stiffness Switchable Hydroplastic Wood with Programmability and Reproducibility. ACS Nano.

[142] Zhang H., Wang X., Wang Y., Gu Z., Chen L. (2022). Bi-functional water-purification materials derived from natural wood modified TiO2 by photothermal effect and photocatalysis. RSC Adv..

[143] Cui G., Guo X., Deng L. (2024). Preparation strategies of mussel-inspired chitosan-based biomaterials for hemostasis. Front. Pharmacol..

[144] Zhu G., Liu C., Zhang C., Shi J., Mei C., Pan M., Liu Z. (2024). Activating the Room-Temperature Phosphorescence of Organic Dyes through the Confinement Effect of Delignified Wood. ACS Sustain. Chem. Eng..

[145] Ran Y., Li J., Zhang S., Wang J., Huang Y., Wang W., Cao J. (2024). Super-stable modified wood for enhanced autonomous indoor humidity regulation. Chem. Eng. J..

[146] Wang Z., Zhang X.-F., Shu L., Yao J. (2023). Construction of MXene functionalized wood-based hydrogels using ZnCl2 aqueous solution for flexible electronics. J. Mater. Chem. A.

